# Mating pair stabilization mediates bacterial conjugation species specificity

**DOI:** 10.1038/s41564-022-01146-4

**Published:** 2022-06-13

**Authors:** Wen Wen Low, Joshua L. C. Wong, Leticia C. Beltran, Chloe Seddon, Sophia David, Hok-Sau Kwong, Tatiana Bizeau, Fengbin Wang, Alejandro Peña, Tiago R. D. Costa, Bach Pham, Min Chen, Edward H. Egelman, Konstantinos Beis, Gad Frankel

**Affiliations:** 1 MRC Centre for Molecular Microbiology and Infection, Imperial College, London, UK; 2 Department of Life Sciences, Imperial College, London, UK; 3 Department of Biochemistry and Molecular Genetics, University of Virginia, Charlottesville, VA USA; 4 Rutherford Appleton Laboratory, Research Complex at Harwell, Oxfordshire, UK; 5 Centre for Genomic Pathogen Surveillance, Big Data Institute, Li Ka Shing Centre for Health Information and Discovery, University of Oxford, Oxford, UK; 6 Department of Chemistry, University of Massachusetts Amherst, Amherst, MA USA

**Keywords:** Bacterial genes, Bacterial genetics

## Abstract

Bacterial conjugation mediates contact-dependent transfer of DNA from donor to recipient bacteria, thus facilitating the spread of virulence and resistance plasmids. Here we describe how variants of the plasmid-encoded donor outer membrane (OM) protein TraN cooperate with distinct OM receptors in recipients to mediate mating pair stabilization and efficient DNA transfer. We show that TraN from the plasmid pKpQIL (*Klebsiella pneumoniae*) interacts with OmpK36, plasmids from R100-1 (*Shigella flexneri*) and pSLT (*Salmonella* Typhimurium) interact with OmpW, and the prototypical F plasmid (*Escherichia coli*) interacts with OmpA. Cryo-EM analysis revealed that TraN_pKpQIL_ interacts with OmpK36 through the insertion of a β-hairpin in the tip of TraN into a monomer of the OmpK36 porin trimer. Combining bioinformatic analysis with AlphaFold structural predictions, we identified a fourth TraN structural variant that mediates mating pair stabilization by binding OmpF. Accordingly, we devised a classification scheme for TraN homologues on the basis of structural similarity and their associated receptors: TraNα (OmpW), TraNβ (OmpK36), TraNγ (OmpA), TraNδ (OmpF). These TraN-OM receptor pairings have real-world implications as they reflect the distribution of resistance plasmids within clinical Enterobacteriaceae isolates, demonstrating the importance of mating pair stabilization in mediating conjugation species specificity. These findings will allow us to predict the distribution of emerging resistance plasmids in high-risk bacterial pathogens.

## Abstract (web summary)

Combining conjugation and structural analyses, the authors show that TraN-OMP pairings determine bacterial conjugation species specificity, with implications in resistance plasmid distribution within Enterobacteriaceae.

## Main

Conjugative transfer of DNA involves a type IV secretion system (T4SS), the relaxosome and a conjugative pilus^1^. In the prevailing model of conjugation, the pilus extends off the surface of the donor and establishes contact with a recipient^2^. It then retracts^3^, drawing the recipient towards the donor leading to the formation of a tight mating junction through a process termed ‘mating pair stabilization’ (MPS)^4^. Recently, important advancements have been made in elucidating the structure of the T4SS and conjugative pilus from several incompatibility group F (IncF) plasmids^5–7^. However, the mechanism underpinning MPS remains relatively unclear.

IncF plasmids are restricted to the Enterobacteriaceae family^8^, and include the resistance plasmid R100 (*Shigella flexneri*) and the virulence plasmids pMAR7 (enteropathogenic *Escherichia coli*) and pSLT (*Salmonella* Typhimurium)^9–12^. Conjugation studies performed in the 1970s focused on the prototypical F plasmid. Through the isolation of *E. coli* ‘Con^−^ mutants’ defective in F plasmid uptake, the outer membrane (OM) protein OmpA and lipopolysaccharide (LPS) were identified as recipient conjugation factors^13–15^. Notably, mutations in *ompA* affected conjugation of the F plasmid but not R100-1, a derepressed derivative of R100^13^.

OmpA dependency was associated with the plasmid-encoded donor subunit TraN as substitution of *traN* from the F plasmid with *traN* from R100-1 abrogated the conjugation deficiency^16^. TraN is an OM protein containing 22 conserved cysteine residues and is involved in the formation of mating aggregates during MPS^2,17,18^. The N-terminal domain, specifically, mediates specificity for recipient OmpA. Despite compelling genetic evidence that TraN cooperates with OmpA to mediate MPS, attempts to isolate a TraN-OmpA complex were unsuccessful^17^.

While IncF plasmid conjugation has been extensively studied in *E. coli* K12, few studies have investigated conjugation of contemporary antibiotic resistance plasmids found in clinically relevant pathogens. *Klebsiella pneumoniae* is a Gram-negative pathogen frequently associated with antimicrobial resistance^19^. Carbapenem resistant *K. pneumoniae* (CRKP) isolates often carry carbapenemase-encoding plasmids, including the *K. pneumoniae* carbapenemase (KPC)-encoding plasmid pKpQIL, which expresses conjugation machinery similar to that found on F and R100-1^20,21^. pKpQIL is particularly closely associated with clinical isolates of the globally pervasive *K. pneumoniae* sequence type ST258^22,23^. While temperature and mating substrate moderately affect conjugative transfer^24^, the role of recipient cells during pKpQIL conjugation is unclear.

In addition to resistance plasmids, mutations affecting the *K. pneumoniae* OM trimeric channel-forming porins OmpK35 and OmpK36 (homologues of OmpF and OmpC in *E. coli*, respectively) also contribute to reduced carbapenem susceptibility^25^. The selectivity of the porins is conferred by loop 3 (L3) which extends into the barrel of the porin, forming a constriction point within the channel^26^. In ST258 CRKP isolates, the introduction of a premature stop codon in *ompK35* is ubiquitous, leading to loss of functional OmpK35^27^. Moreover, many ST258 isolates express OmpK36 variants containing L3 insertions that are not present in OmpK36 from the reference laboratory strain ATCC 43816, a derivative of which serves as the wild type (WT) strain in this work^28^. We showed that a glycine-aspartic acid (GD) ST258-associated L3 insertion constricts the channel by 26%, reducing antibiotic diffusion across the OM^29^.

### pKpQIL conjugation is dependent on recipient OmpK36

**Fig. 1:**
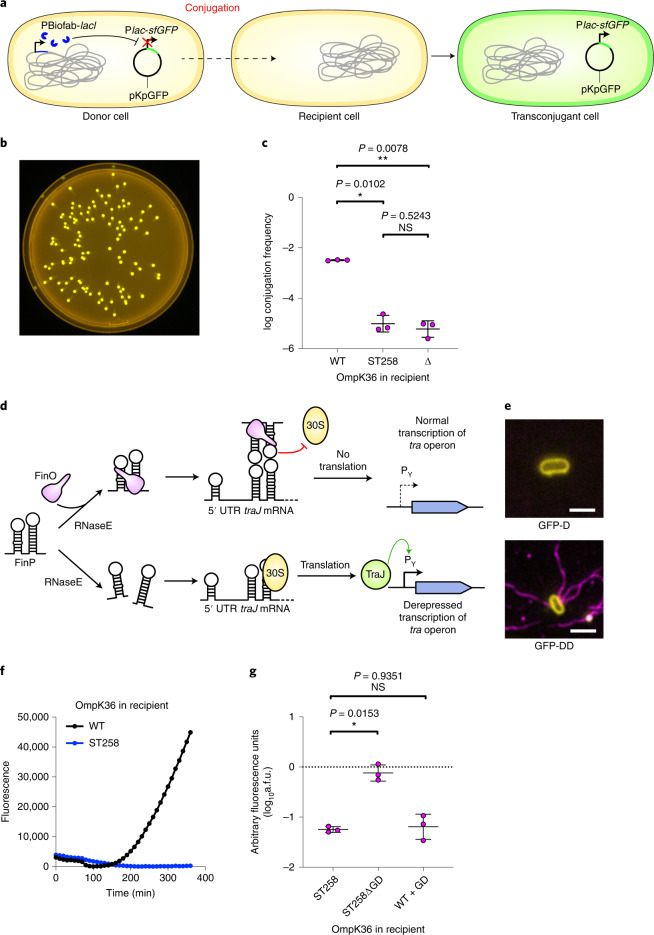
pKpGFP conjugation is dependent on OmpK36 and is disrupted by the L3 GD insertion. **a**,**b**, Schematic (**a**) showing the strategy used to generate the P*lac*-*sfGFP* pKpQIL reporter, pKpGFP. pKpGFP is carried in a donor that constitutively expresses *lacI*, resulting in *sfGFP* repression. Upon conjugative transfer into recipients that do not carry the synthetic *lacI*, *sfGFP* is expressed in transconjugant cells (**b**). **c**, Conjugation of pKpGFP into recipients expressing 36_ST258_ or Δ*ompK36* were compared to recipients expressing 36_WT_. log conjugation frequency data are presented as mean ± s.d. of three biological repeats, analysed by repeated measures one-way ANOVA with Tukey’s multiple comparison test. **d**, Diagram of the FinOP fertility inhibition system of IncF conjugative plasmids. **e**, The donor strain GFP-DD carrying the derepressed plasmid pKpGFP-D is piliated (magenta, bottom). FM4-64 (yellow) was used to stain the bacterial cell membrane. Piliation was not observed on GFP-D cells carrying pKpGFP (top). Scale bar, 2 μm. **f**, The effect of the GD insertion on pKpGFP-D conjugation was assessed by measuring GFP emission from transconjugants over time. Measurements were recorded every 10 min and a representative graph of fluorescence over time is shown for plasmid uptake in receivers expressing 36_WT_ or 36_ST258_. **g**, RTCS data, shown as a.f.u., are presented as mean ± s.d. of three biological repeats, analysed by repeated measures one-way ANOVA with Dunnett’s multiple comparison test comparing to the OmpK36_ST258_-expressing recipient. NS, not significant. Source data

The aim of this study was to determine whether ST258-associated porins affect pKpQIL conjugation. As *ompK35* is a pseudogene in these isolates, we assessed the role of OmpK36 in the absence of OmpK35 expression (Supplementary Table 1). We generated a reporter pKpQIL called pKpGFP (Fig. 1a), which expresses superfolder green fluorescent protein (sfGFP) under control of a *lac* promoter (P*lac*), allowing for quantification of conjugation frequency by selective plating or fluorescence assays. We then engineered a *K. pneumoniae* donor that constitutively expresses LacI for repression of *sfGFP* expression. pKpGFP was transferred into this strain, which we named GFP donor (GFP-D) (Supplementary Table 1). Upon conjugative transfer of pKpGFP from GFP-D into a *K. pneumoniae* recipient that does not constitutively express LacI, sfGFP is expressed (Fig. 1b).

**Extended Data Fig. 1:**
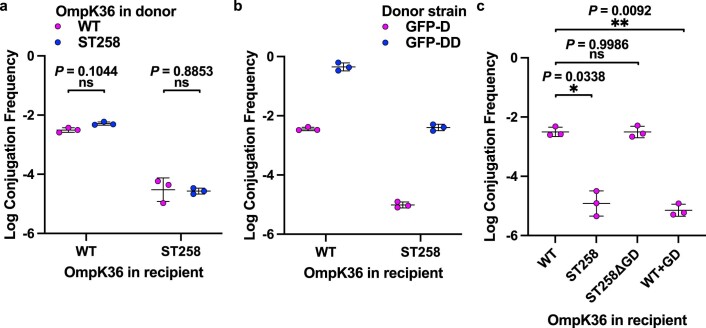
Characterisation of OmpK36-dependence and pKpGFP derepression. **a** The effect of the OmpK36 mutations on conjugation is specific to the recipient. **b** Deletion of *finO* increases transfer of pKpGFPΔ*finO* compared to pKpGFP; the difference in log conjugation frequency due to the ST258 mutations in recipients is maintained. **c** Conjugation frequency of pKpGFP into strains expressing different OmpK36 isoforms. Log conjugation frequency is presented as the mean ± SD of three biological repeats analyzed by multiple paired *t* tests with Holm-Šídák correction in (**a**) and by repeated measures one-way ANOVA with Dunnett’s multiple comparison test in (**c**). ns, not significant. Source data

We compared the conjugation efficiency of pKpGFP from GFP-D into recipients expressing WT OmpK36 (36_WT_), ST258 OmpK36 containing the L3 GD insertion (36_ST258_) or missing *ompK36* (Δ*ompK36*). A similar reduction in pKpGFP conjugation frequency into 36_ST258_ and Δ*ompK36* recipients was observed compared with a recipient expressing 36_WT_ (Fig. 1c). Next we determined whether the impact of 36_ST258_ on pKpGFP conjugation is restricted to the recipient by comparing plasmid transfer between GFP-D expressing either 36_WT_ or 36_ST258_. Both donors exhibited similar conjugation frequencies (Extended Data Fig. 1a), suggesting that 36_ST258_ impairs conjugation when expressed specifically in the recipient.

We next determined the mechanism by which 36_ST258_ reduces conjugative uptake of pKpGFP. *K. pneumoniae* expressing 36_ST258_ are less susceptible to carbapenems compared with those expressing 36_WT_^25,29^. To overcome this, we used pKpGFP to develop a high throughput, real-time conjugation system (RTCS) assay, to quantify conjugation frequency in the absence of selective pressure by measuring temporal fluorescence emission from conjugation mixtures. However, using the GFP-D reporter in RTCS, we did not detect an increase in fluorescence even with *K. pneumoniae* recipients expressing OmpK36_WT_. This suggested that RTCS was not sensitive enough to detect transfer of pKpGFP (Fig. 1c). To increase conjugation efficiency, we deleted the conjugation inhibitor gene *finO* from pKpGFP to generate a derepressed variant of pKpGFP, pKpGFP-D (Fig. 1d)^30^. pKpGFP-D was transferred into the strain overexpressing LacI to generate the GFP-derepressed donor (GFP-DD).

We stained GFP-D and GFP-DD with rat polyclonal antiserum raised against the intact conjugation pilus. Only GFP-DD bacteria were piliated, which confirmed that deleting *finO* results in derepression of transfer genes (Fig. 1e). Transfer of pKpGFP-D into recipients was also observed by live microscopy (Supplementary Video). Comparing the conjugation frequency of pKpGFP and pKpGFP-D revealed an overall 2-log-fold increase in transfer of pKpGFP-D (Extended Data Fig. 1b). Importantly, the relative difference in conjugation frequency into recipients expressing 36_WT_ and 36_ST258_ seen using GFP-D was maintained using GFP-DD. When total fluorescence outputs from conjugation mixtures containing GFP-DD were measured in RTCS, an increase in fluorescence was detected as early as 150 min for recipients expressing 36_WT_ but not 36_ST258_ (Fig. 1f).

We used RTCS to determine whether the L3 GD insertion present in 36_ST258_ affects conjugative uptake of pKpGFP. Deleting the GD insertion (36_ST258ΔGD_) led to a significant increase in a.f.u. compared with those expressing 36_ST258_ (Fig. 1g). This effect could be reconstituted by introducing the GD insertion into OmpK36_WT_ (36_WT+GD_) and was validated by selective plating (Extended Data Fig. 1c). These results suggest that the GD insertion reduces conjugative uptake of pKpGFP-D, potentially due to pore constriction^29^.

### TraN homologues cooperate with distinct recipient OM proteins

**Fig. 2:**
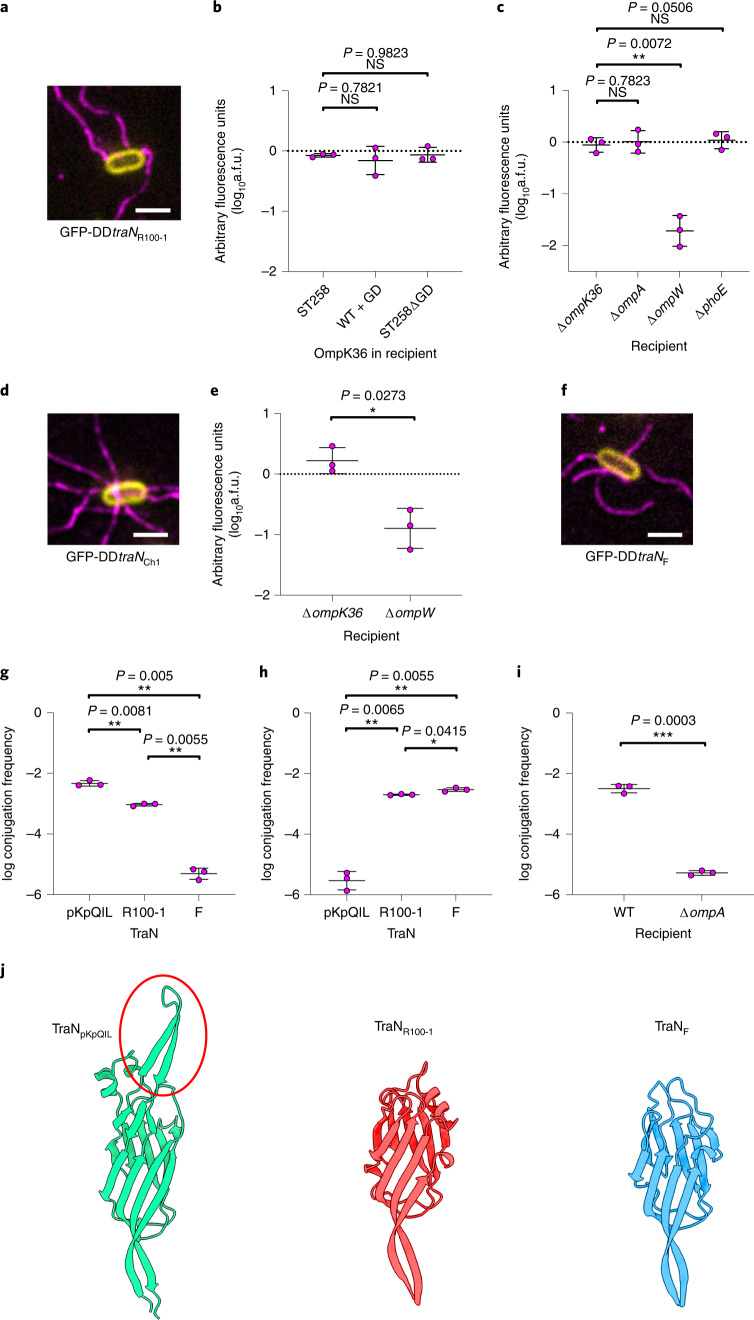
Structural differences in TraN mediate specificity for recipient OM receptors. **a**, Substitution of pKpQIL *traN* with *traN* from R100-1 does not affect piliation (magenta) as seen by immunofluorescence microscopy of donor cells carrying pKpGFP-D*traN*_R100-1_ (GFP-DD*traN*_R100-1_). Scale bar, 2 μm. **b**, RTCS was used to assess the effect of the L3 GD insertion on pKpGFP-D*traN*_R100-1_ conjugation. **c**, pKpGFP-D*traN*_R100-1_ conjugation into a panel of isogenic recipients was assessed using RTCS. A.f.u. data are presented as mean ± s.d. of three biological repeats, analysed by repeated measures one-way ANOVA with Dunnett’s multiple comparison test comparing to the OmpK36_ST258_-expressing recipient in (**b**) and to the Δ*ompK36* recipient in (**c**). **d**, Donor cells carrying pKpGFP-D*traN*_Ch1_ are piliated. Scale bar, 2 μm. **e**, Recipient OM protein dependency during pKpGFP-D*traN*_Ch1_ conjugation was determined with RTCS. A.f.u. data are presented as mean ± s.d. of three biological repeats, analysed by a two-sided paired *t*-test. **f**, The GFP-D*traN*_F_ donor is piliated. Scale bar, 2 μm. **g**,**h**, The log conjugation frequency of pKpGFP into WT *K. pneumoniae* (**g**) and *E. coli* MG1655 (**h**) recipients as a function of different TraN homologues was measured. **i**, log conjugation frequency of GFP-D*traN*_F_ into WT and Δ*ompA* recipients was compared. log conjugation frequency data are presented as mean ± s.d. of three biological repeats, analysed by repeated measures ANOVA and Tukey’s multiple comparison test in **g** and **h**, and by a two-sided paired *t*-test in **i**. **j**, Zoomed in cartoon representation of the tip region from each TraN variant showing a predicted β-hairpin structure that is unique to TraN_pKpQIL_ (circled). Source data

**Extended Data Fig. 2:**
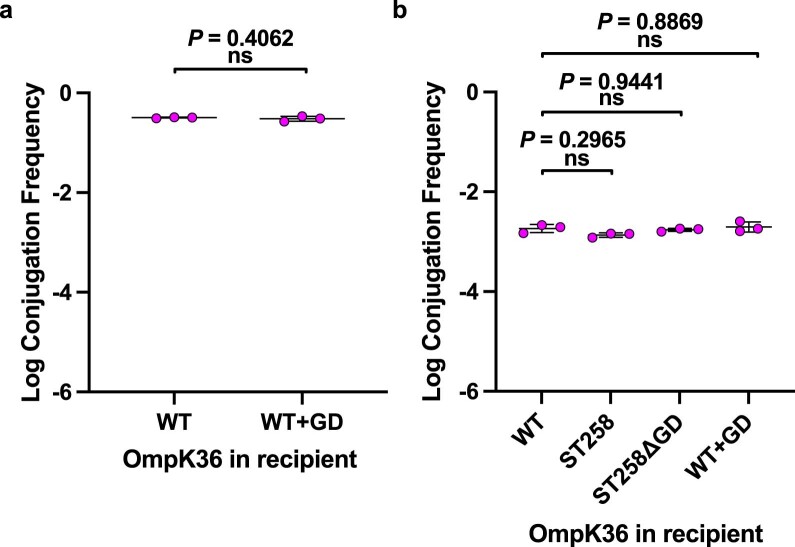
OmpK36-dependency in R100-1 conjugation. **a** R100-1 conjugates similarly into recipients expressing OmpK36_WT_ or OmpK36_WT+GD_. **b** Conjugation frequency of pKpGFP expressing TraN_R100-1_ into strains expressing different OmpK36 isoforms. Log conjugation frequency is presented as the mean ± SD of three biological repeats analyzed by a two-sided paired *t* test in (**a**) and by a repeated measures one-way ANOVA with Dunnett’s multiple comparison test in (**b**). ns, not significant. Source data

**Extended Data Fig. 3:**
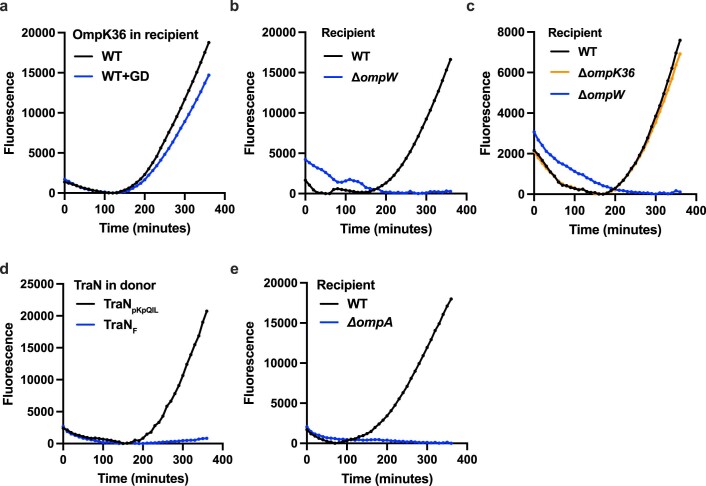
Temporal, real-time measurement of conjugation using the RTCS. **a** Substitution of *traN* in pKpGFP-D with *traN* from R100-1 abrogates the effect of the L3 GD insertion in OmpK36 on conjugation. **b** pKpGFP-D*traN*_R100-1_ conjugation is dependent on OmpW in the recipient. **c** A chimeric TraN (TraN_Ch1_) which contains the variable domain from TraN_R100-1_ and the N- and C-termini from TraN_pKpQIL_ confers OmpW dependency during conjugation. **d** pKpGFP-D expressing *traN* from F does not conjugate efficiently into *K. pneumoniae* recipients. **e** pKpGFP-D*traN*_F_ conjugation is dependent on OmpA expressed in *E. coli* recipients. Fluorescence emission was measured every 10 m and normalized to the minimum emission recorded from each conjugation mixture over the 6 h incubation period. Graphs are representative of three biological replicates. Source data

As *E. coli* OmpA dependency in F plasmid conjugation could be circumvented by substituting its *traN* with *traN* from R100-1^16^, we hypothesized that OmpK36 dependency seen in pKpQIL is similarly mediated by TraN. We first investigated whether R100-1 conjugation is OmpK36 dependent and observed no significant difference into *K. pneumoniae* recipients expressing either 36_WT_ or 36_WT+GD_ (Extended Data Fig. 2a). We then substituted *traN* from both pKpGFP and pKpGFP-D with *traN* of R100-1 (*traN*_R100-1_); immunofluorescence staining showed that donor cells carrying pKpGFP-D*traN*_R100-1_ (GFP-DD*traN*_R100-1_) are piliated (Fig. 2a). Using GFP-DD*traN*_R100-1_ in RTCS revealed that conjugation was no longer affected by the GD insertion in OmpK36 (Fig. 2b and Extended Data Fig. 3a). This was validated by selective plating (Extended Data Fig. 2b). These results show that OmpK36-dependency during conjugation is plasmid specific and mediated by TraN.

**Extended Data Fig. 4:**
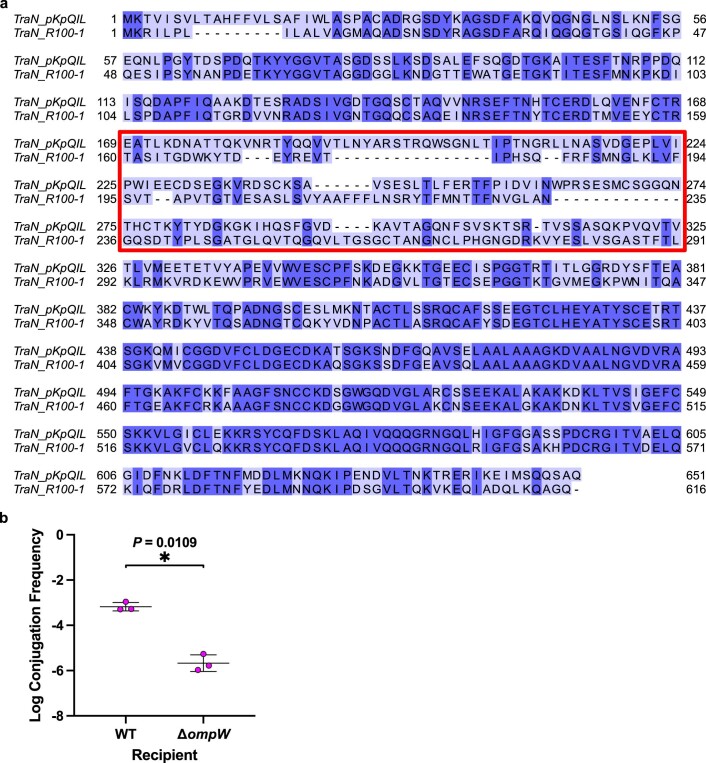
The variable domain of TraN determines recipient OM protein dependency. **a** The domain which was substituted to generate the chimeric TraN (TraN_Ch1_) is highlighted in red. Alignment was generated using Clustal Omega and visualized with Jalview 2.11.1.4. **b** Conjugation frequency of pKpGFP expressing TraN_Ch1_ into WT and Δ*ompW* recipients. Log conjugation frequency is presented as the mean ± SD of three biological repeats analyzed by a two-sided paired *t* test. Source data

We hypothesized that TraN_R100-1_ cooperates with another OM protein in *K. pneumoniae* and identified PhoE^31^ and OmpW^32^, which are conserved in Enterobacteriaceae species, as candidates and generated Δ*phoE* and Δ*ompW* mutants. Using RTCS to assess plasmid transfer into Δ*phoE* and Δ*ompW* alongside Δ*ompK36* and Δ*ompA* recipients revealed significantly lower conjugation frequency specifically into the Δ*ompW* recipient (Fig. 2c and Extended Data Fig. 3b). To determine which domain of TraN mediates specificity, we generated a chimaeric *traN* (*traN*_Ch1_) by substituting amino acid residues from the variable N-terminal domain of TraN_pKpQIL_ with corresponding residues from TraN_R100-1_ (Extended Data Fig. 4a). Donor cells expressing *traN*_Ch1_ (GFP-DD*traN*_Ch1_) were piliated (Fig. 2d) and facilitated efficient conjugation into the Δ*ompK36* but not Δ*ompW* recipients (Fig. 2e and Extended Data Figs. 3c and 4b). These results show that TraN mediates efficient conjugation via specific pairings of TraN_R100-1_ with OmpW and TraN_pKpQIL_ with OmpK36, with specificity conferred by the variable N-terminal domain.

We next sought to validate that TraN from F mediates dependency on OmpA. We substituted *traN* from pKpGFP and pKpGFP-D with *traN* from F (*traN*_F_) to generate pKpGFP*traN*_F_ and pKpGFP-D*traN*_F_, respectively. Donors carrying pKpGFP-D*traN*_F_ (GFP-DD*traN*_F_) were piliated (Fig. 2f); however, we observed no increase in fluorescence with the WT recipient compared with the GFP-DD donor (Extended Data Fig. 3d). Nevertheless, using donors carrying pKpGFP*traN*_F_ (GFP-D*traN*_F_) in selection-based assays (which are more sensitive than RTCS) with WT *K. pneumoniae* recipients, we detected transconjugants, confirming the expression of functional conjugation machinery (Fig. 2g). However, compared with donor strains expressing either TraN_pKpQIL_ or TraN_R100-1_, the conjugation frequency associated with the TraN_F_-expressing donor was significantly lower. As OmpA was first proposed as a receptor for TraN_F_ in *E. coli*, we hypothesized that the *K. pneumoniae* homologue of OmpA is not recognized by TraN_F_. Therefore, we repeated the experiment using *E. coli* MG1655 recipients. We observed that conjugation of pKpGFP expressing either TraN_F_ or TraN_R100-1_ was significantly higher than that of the TraN_pKpQIL_-expressing plasmid (Fig. 2h). This suggests that TraN_F_, but not TraN_pKpQIL_, cooperates with an *E. coli* OM protein. In contrast, TraN_R100-1_ facilitates efficient conjugation into both *K. pneumoniae* and *E. coli*, which suggests that it recognizes OmpW in both bacterial species^32^. TraN_F_ was confirmed to cooperate with *E. coli* OmpA as we observed a significant decrease in conjugation frequency of pKpGFP*traN*_F_ into an MG1655Δ*ompA* compared with the WT recipient (Fig. 2i and Extended Data Fig. 3e).

**Extended Data Fig. 5:**
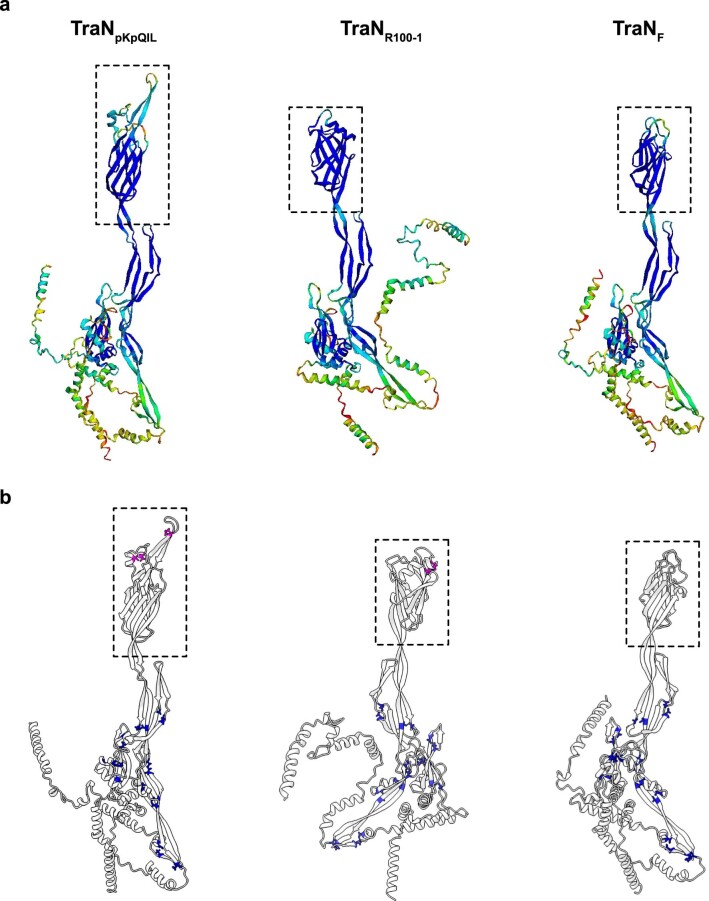
Comparison of predicted TraN structures. **a** The structures of TraN from pKpQIL, R100-1 and F were predicted using AlphaFold. TraN is shown in ribbon and coloured by pLDDT score: red (very low, <50 %), yellow (low, 60 %), green (OK, 70 %), light blue (confident, 80 %) and dark blue (very high, >90 %). The predicted structures showed a high degree of structural similarity in the core of the protein whereas the ‘tip’ (highlighted in dashed boxes) is variable. **b** Cysteines in the predicted structures of TraN_pKpQIL_, TraN_R100-1_ and TraN_F_ are highlighted. Distance measurements between cysteine residues were performed in ChimeraX and residues which can form disulphide bonds are listed in Supplementary Table 7.

To understand how the different TraN variants mediate OM receptor specificity, we used AlphaFold^33^ to generate predicted structures for TraN_pKpQIL_, TraN_R100-1_ and TraN_F_ (Extended Data Fig. 5a). The predicted models show a high overall predicted local-distance difference test (pLDDT) score, providing confidence in the structural variation seen between the different alleles. Each variant appears to contain an amphipathic alpha-helix that can potentially anchor it to the outer leaflet of the OM. The overall structure contains an extended N-terminal domain consisting mostly of β-sheets linked to a β-sandwich domain that we refer to as the ‘tip’, while the C-terminal domain is a mix of α-helices and β-sheets that fold back and form intradomain contacts with the N-terminal domain. From the predicted structures, all cysteine residues in each TraN variant could be engaged in intramolecular disulfide bonds (Extended Data Fig. 5b and Supplementary Table 7). Interestingly, structural differences are mainly seen in the ‘tip’ region of the protein, which corresponds to the variable region of the TraN sequences, consistent with their OM specificity observed during functional conjugation assays (Fig. 2j).

### TraN_pKpQIL_ forms a complex with OmpK36

**Extended Data Fig. 6:**
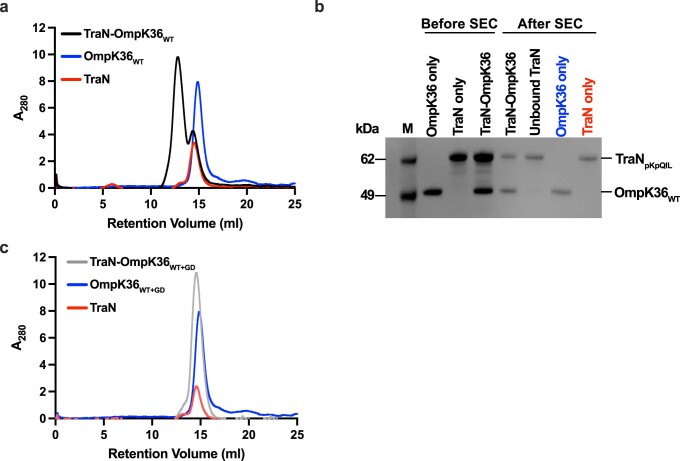
Size exclusion chromatography of TraN_pKpQIL_ and OmpK36_WT_. **a** Incubation of TraN with OmpK36_WT_ results in the formation of a stable complex as shown by SEC. Comparison of the chromatograms indicates the clear shift for the TraN-OmpK36 complex (black) compared to free TraN (red) and OmpK36_WT_ (blue). **b** The complex formation was further verified by SDS-PAGE and it shows that the peak at 12.8 mL contains both TraN and OmpK36_WT_. SDS-PAGE analysis includes samples before and after SEC with label colors corresponding to the chromatogram peaks; Lane M, marker in kDa. **c** Incubation of TraN with OmpK36_WT+GD_ results in no complex formation. Comparison of the chromatograms indicates that there is no shift in the retention volume for the TraN-OmpK36_WT+GD_ sample (grey) compared to free TraN (red) and OmpK36_WT+GD_ (blue). Source data

To determine the molecular basis of TraN_pKpQIL_-mediated conjugation dependency on OmpK36, we purified TraN_pKpQIL_ (68 kDa) and OmpK36_WT_ (~120 kDa (trimer)) and tested their ability to form a complex by size exclusion chromatography (SEC). A clear shift in the retention volume of the TraN-OmpK36 sample (12.8 ml) was observed compared with the individual retention volumes of TraN_pKpQIL_ (14.5 ml) and OmpK36 (14.9 ml), which indicates the formation of a stable TraN-OmpK36 complex (Extended Data Fig. 6a). Complex formation was confirmed by SDS–PAGE analysis (Extended Data Fig. 6b). In contrast, no shift was observed when TraN was mixed with OmpK36_WT+GD_ (Extended Data Fig. 6c), suggesting that the GD insertion impairs complex formation.

**Fig. 3:**
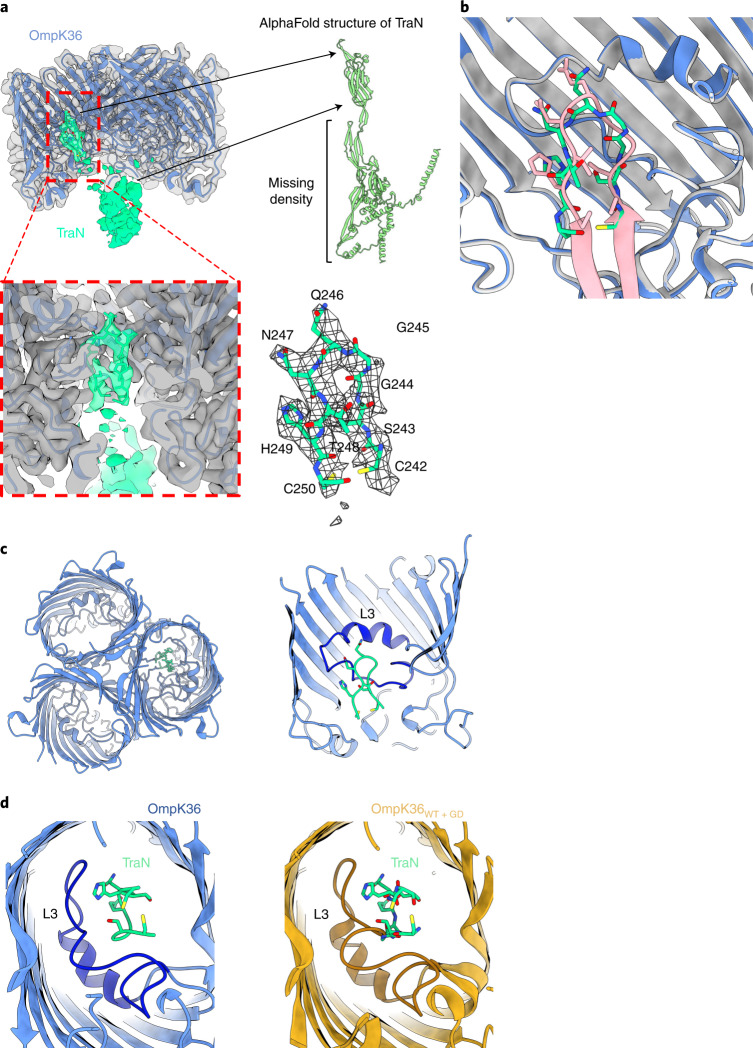
Cryo-EM structure of the TraN-OmpK36_WT_ complex. **a**, Cryo-EM reconstruction of the complex at 2.6 Å resolution. The reconstruction (left) shows Coulomb potential density for the OmpK36 trimer (transparent grey density) and TraN (green density). The front view of the reconstruction perpendicular to the OM has been omitted to reveal the TraN density within the channel. The OmpK36 and TraN atomic models have been fitted inside the reconstruction. The predicted AlphaFold structure of TraN is shown as cartoons (right). The TraN density accounts for the β-hairpin and β-sandwich domains; however, density is missing for the remainder of the predicted AlphaFold structure. Close-up view of the TraN β-hairpin model fitted inside the density (red box and bottom right). **b**, AlphaFold predicted a very similar complex formation with the β-hairpin inserting inside the OmpK36 pore. The cryo-EM complex (OmpK36 in blue/TraN in green) and AlphaFold model (OmpK36 in grey/TraN in pink) can be superimposed without any major deviations. **c**, Top view of OmpK36 (blue) interaction with the β-hairpin of TraN (green) (left). TraN inserts halfway inside the OmpK36 channel to interact with L3 (right); the front face of the OmpK36 barrel has been omitted for clarity. **d**, The conformation of the OmpK36_WT_ L3 can accommodate the TraN β-hairpin (left), whereas the L3 GD insertion results in steric clashes (right) that prevent complex formation.

**Extended Data Fig. 7:**
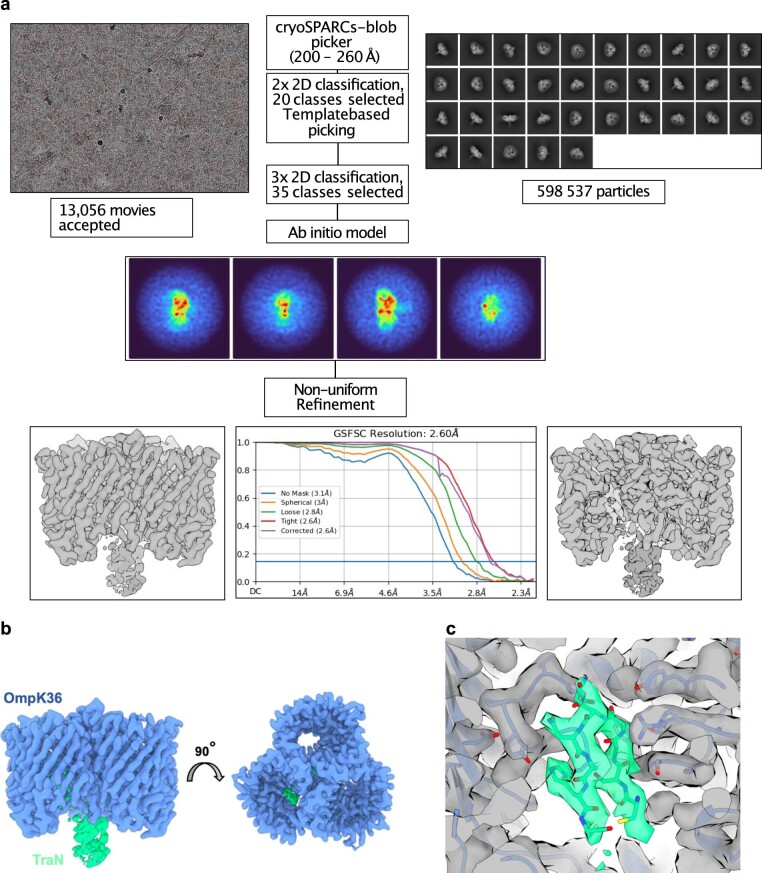
Cryo-EM and single-particle analysis of OmpK36-TraN. **a** Overview of the image processing workflow done in cryoSPARC v3.2.0. 13,056 movies were selected based on estimated resolution, defocus, ice thickness, and full-frame motion. Accepted micrographs were subjected to automated particle picking ‘blob picking’ and 2x 2D classification to obtain template references. Template-based picking followed by 3x 2D classification was completed, resulting in 508, 537 particles. Particles were then used for Ab initio reconstruction; the best output reconstruction generated by Ab initio reconstruction was further refined using Non^−^uniform Refinement. The results of Non-uniform refinement yielded a reconstruction with an estimated resolution of 2.6 Å. **b** Coulomb potential density for the OmpK36 trimer (blue density) and TraN (green density). **c** Close up view of the density for the OmpK36 and TraN ‘tip’ side chains.

The structural basis of the TraN-OmpK36 interaction was assessed by cryo-electron microscopy (cryo-EM), which showed discrete TraN-OmpK36 complexes. However, TraN was mainly disordered and exhibited an occupancy of less than one molecule for each OmpK36 trimer. A three-dimensional (3D) reconstruction for the complex with an overall resolution of 2.6 Å using the map:map approach was generated with density for both the OmpK36 trimer and TraN (Fig. 3a and Extended Data Fig. 7). The crystal structure of the trimeric OmpK36 (PDB 6RD3)^29^ was placed inside the density with minimal rebuilding, mostly amino acid rotamers and correcting for Ramachandran outliers on the basis of electron density and bond strains; the OmpK36 crystal and cryo-EM structures display a root-mean-square deviation (rmsd) of 0.6 Å over 480 C_α_ atoms. The additional density below OmpK36 was assigned to TraN (Fig. 3a and Extended Data Fig. 7) and it extends into the channel of one subunit of the trimeric porin. The TraN density is weak, present at a low threshold and featureless. Therefore, we decided not to build the TraN model in this density. The density inside the pore is better defined, showing a loop-shaped appearance with side chains; using the AlphaFold model for TraN_pKpQIL_, that density corresponds to the predicted β-hairpin of the TraN_pKpQIL_ ‘tip’ (Figs. 2j and 3a). Further evidence that the density inside the pore corresponds to the TraN ‘tip’ was provided by generating an ab initio complex, which predicted a nearly identical OmpK36-TraN complex as the cryo-EM structure, with an rmsd of 0.45 Å over 480 C_α_ OmpK36 atoms and an rmsd of 1.2 Å for the 9 C_α_ β-hairpin atoms (Fig. 3b).

TraN inserts into the OmpK36 pore from its extracellular side and reaches halfway through the channel to L3 (Fig. 3c). The binding/recognition of the TraN β-hairpin is mostly mediated by interactions with L3 of OmpK36. Structural comparison was performed to investigate the disruption in complex formation caused by the L3 GD insertion (Fig. 3d). Using the TraN-OmpK36_WT_ model as a reference, the structure for OmpK36_WT+GD_ (PDB 6RCK)^29^ was superimposed onto the complex (Fig. 3d). This showed a clash between Gly115 and Asp114 from L3 of OmpK36, and Ser243’ and Gly244’ of the TraN β-hairpin (Fig. 3d). These clashes are anticipated to destabilize the OmpK36 and TraN interaction, lowering the affinity of TraN for OmpK36_WT+GD_.

### TraN-OMP interactions influence plasmid host distribution

**Extended Data Fig. 8:**
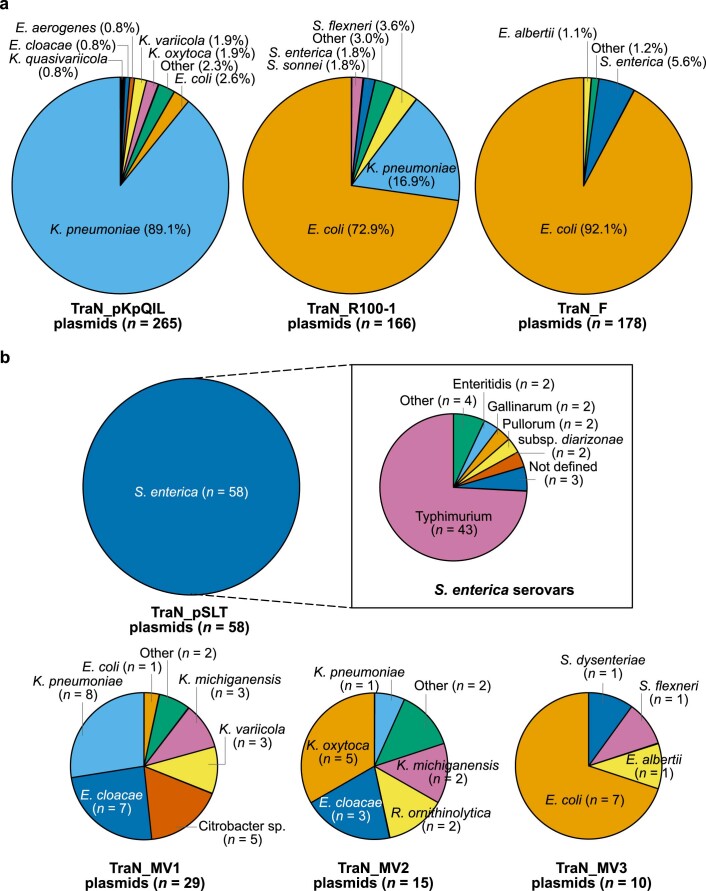
Plasmid host species distribution for TraN sequence variants. **a** tBlastn was used to identify IncF conjugative plasmids which express TraN sharing at least 90% amino acid similarity to TraN expressed from pKpQIL, R100-1 or F. The host species distribution is shown for plasmids carrying these TraN variants. Where only one plasmid was associated with a species, it was categorized as ‘Others’. **b** Host species distribution of other TraN variants in at least 10 plasmids are shown. For plasmids carrying a TraN sharing at least 90% similarity to TraN from the *S*. Typhimurium virulence plasmid, pSLT, the associated *S. enterica* serovars are shown in the insert.

We next investigated the real-world implications of TraN-mediated species-specific transfer of plasmids by analysing TraN sequences from a dataset of plasmids retrieved from GenBank in 2018^34^. Using the Plascad tool for plasmid classification^34^ and the NCBI Taxonomy database to determine the bacterial host family, we identified 824 predicted conjugative IncF plasmids from Enterobacteriaceae isolates (Supplementary Table 2). Using tBLASTn^35^, we found that of these plasmids, 265 (32.2%), 166 (20.1%) and 178 (21.6%) contained *traN* genes encoding proteins with ≥90% amino acid similarity to those found in pKpQIL, R100-1 and F, respectively. In total, these variants account for 74% of the 824 plasmids examined. Importantly, plasmids carrying a similar *traN* were found in a small number of species, including one single dominant species. Notably, 89.1% of *traN*_pKpQIL_ plasmids are from *K. pneumoniae*, while 92.1% of *traN*_F_ plasmids were found in *E. coli* (Extended Data Fig. 8a). These findings align with our in vitro data and suggest that TraN-mediated species specificity during conjugation may influence plasmid host range. Finally, while 72.9% of *traN*_R100-1_ plasmids were found in *E. coli*, a substantial proportion (16.9%) were recovered from *K. pneumoniae* isolates, supporting our findings that this TraN variant facilitates MPS in both bacterial species.

Phylogenetic trees were constructed using the different *traN* nucleotide sequences and are available to view on Microreact: TraN_pKpQIL_, TraN_R100-1_, TraN_F_. Among those with the same gene type, there was no correlation between the phylogeny and the plasmid host, suggesting that the plasmids were largely acquired via horizontal gene transfer as opposed to clonal expansion within a species following an initial conjugative event.

We analysed the remaining 215 plasmids for annotated *traN* sequences and identified 4 other variants found in at least 10 sequenced plasmids (Extended Data Fig. 8b). One variant, which aligned with *traN* from the *S*. Typhimurium virulence plasmid, pSLT (accession ID: AE006471.2), was found exclusively in *Salmonella enterica*, with varied distribution within serovars of this species. The 3 remaining variants were not associated with well-known plasmids. Thus, we assigned these plasmids to 1 of 3 ‘minor variant’ groups (MV1–3) represented by *traN* sequences from NZ_CP016763.1 (MV1), AP014954.1 (MV2) and NZ_CP023348.1 (MV3). MV1 and MV2 plasmids did not appear to associate with a single dominant species, while MV3 plasmids were predominantly associated with *E. coli* hosts.

**Extended Data Fig. 9:**
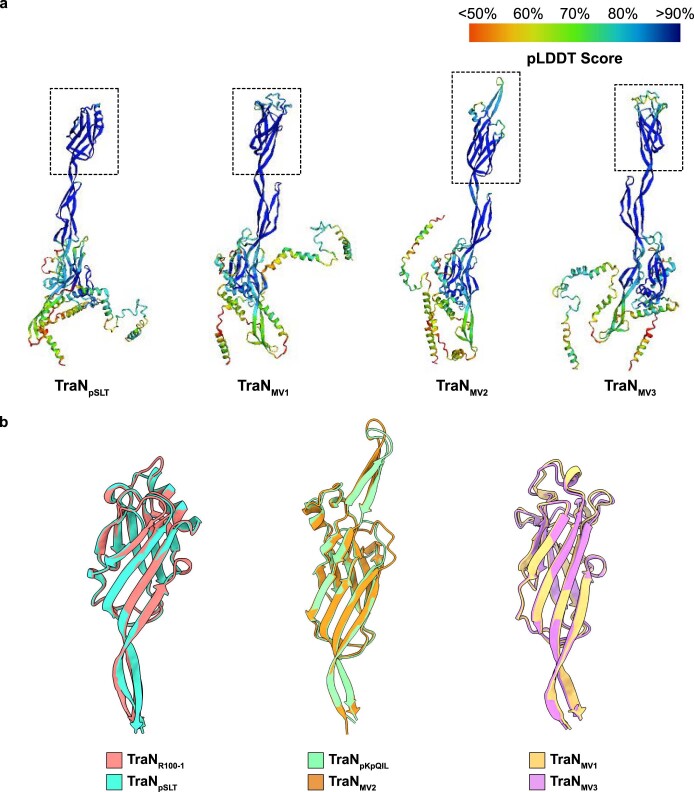
Structural comparison of additional TraN variants. **a** The structures of the additional TraN variants were predicted using AlphaFold. TraN is shown in ribbon and coloured by pLDDT score: red (very low, <50 %), yellow (low, 60 %), green (OK, 70 %), light blue (confident, 80 %) and dark blue (very high, >90 %). The ‘tip’ regions have been highlighted in the dashed box. **b** Predicted tip structures of TraN_R100-1_ and TraN_pSLT_, TraN_pKpQIL_ and TraN_MV2_, and TraN_MV1_ and TraN_MV3_ were superimposed in ChimeraX.

**Extended Data Fig. 10:**
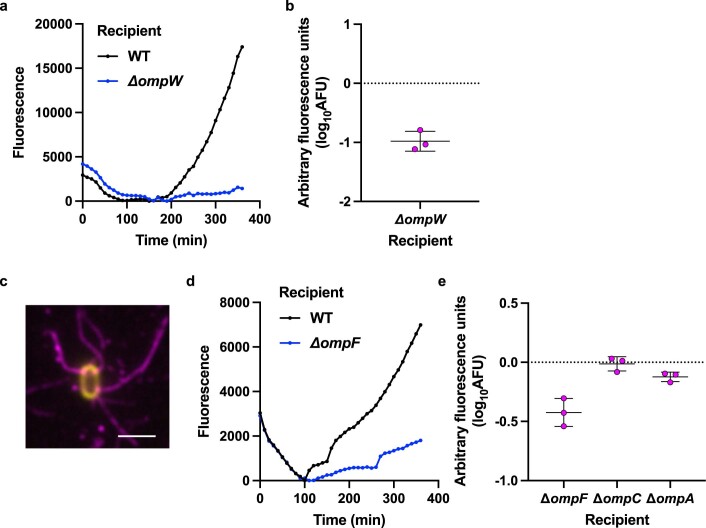
TraN_pSLT_ and TraN_MV1_ receptor specificity. **a** Representative graph showing fluorescence emission over time from pKpGFP-D*traN*_Ch2_ into WT and Δ*ompW* recipients. **b** Arbitrary fluorescence units (AFU) were calculated for the Δ*ompW* recipient as log_10_ (fluorescence_Δ*ompW*_/fluorescence_WT_). **c** GFP-DD*traN*_Ch3_ cells are piliated. The bacterial cell membrane is shown in yellow and conjugative pili are shown in magenta. Scale bar = 2 μm. **d** Representative graph of fluorescence emission over time from pKpGFP-D*traN*_Ch3_ into *E. coli* MG1655 WT and Δ*ompF* recipients. **e** Arbitrary fluorescence units (AFU) were calculated for the Δ*ompF*, Δ*ompC* and Δ*ompA* recipients as log_10_ (fluorescence_X_/fluorescence_WT_) where X is each recipient strain. AFU data in (**b**) and (**e**) is presented as the mean ± SD of three biological repeats. Source data

We used AlphaFold to generate predicted structures for TraN encoded by pLST and the MV1–3 reference plasmids (Extended Data Fig. 9a). Despite sharing less than 90% sequence similarity, the tip structures of TraN_R100-1_ and TraN_pSLT_, TraN_pKpQIL_ and TraN_MV2_, and TraN_MV1_ and TraN_MV3_ are superimposable (Extended Data Fig. 9b). On the basis of these observations, we hypothesized that structurally similar tips recognize the same recipient OM proteins. Thus, we generated a chimaeric TraN containing the TraN_pSLT_ tip (TraN_Ch2_), with functional studies revealing that it mediates OmpW dependency (Extended Data Fig. 10a,b). We next generated a third TraN chimaera (TraN_Ch3_) expressing the tip region from TraN_MV1_. Donors carrying pKpGFP-D*traN*_Ch3_ were piliated (Extended Data Fig. 10c). Testing conjugation of pKpGFP-D*traN*_Ch3_ into a panel of *E. coli* MG1655 recipients lacking OmpA, OmpC or OmpF revealed that TraN_MV1_ cooperates with OmpF (Extended Data Fig. 10d,e).

**Fig. 4:**
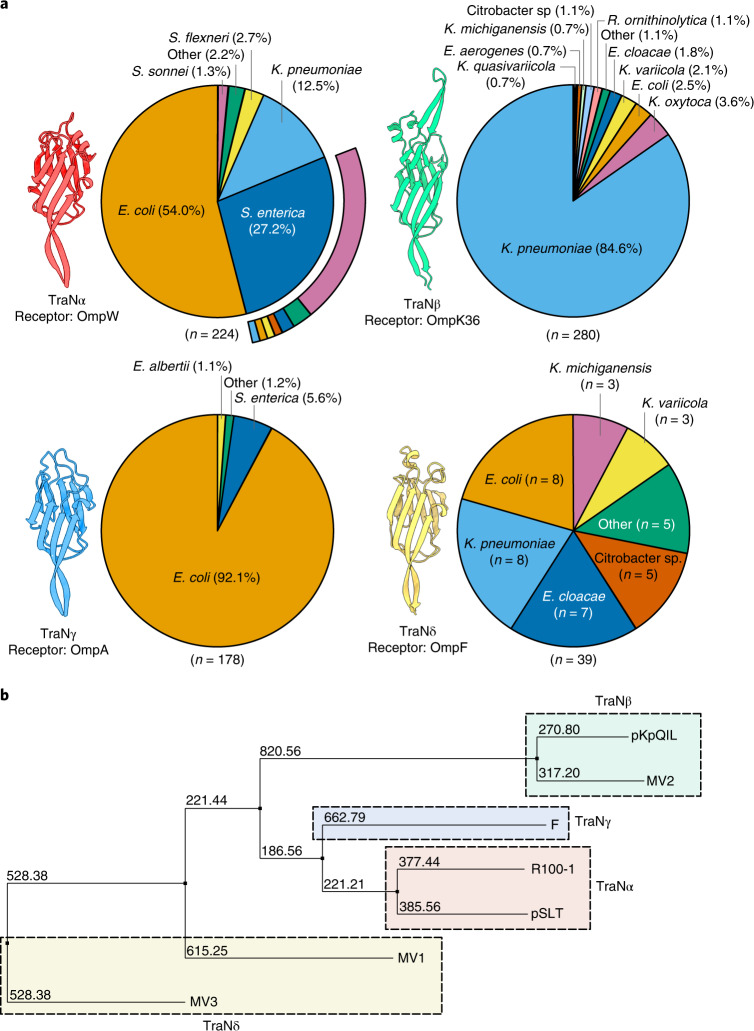
Plasmids encoding structurally similar TraN variants display host species specificity. **a**, TraN sequence variants were classified according to structural similarity of the tip region as follows: TraNα (TraN_R100-1_ and TraN_pSLT_), TraNβ (TraN_pKpQIL_ and TraN_MV2_), TraNγ (TraN_F_), TraNδ (TraN_MV1_ and TraN_MV3_). Receptors for each group are indicated. The pie charts illustrate the host distribution of plasmids expressing each of the structural variants. Where only one plasmid was associated with a species, it was categorized as ‘Other’. The serovar breakdown for *S. enterica* strains carrying TraNα is shown in the outer ring, with the colours corresponding to the insert in Extended Data Fig. 8. **b**, Phylogenetic tree of TraN sequence variants from the seven reference plasmids. Dashed boxes indicate variants assigned to TraN groups determined through structural comparison and receptor specificity.

These findings suggest that tip structure, rather than sequence similarity, determines TraN specificity for recipient OM proteins. Accordingly, we classified the 7 identified variants on the basis of structural similarity into 4 groups denoted TraNα, TraNβ, TraNγ and TraNδ (Fig. 4a). Phylogenetic analysis of the TraN sequences showed clustering, which aligns with our classification system (Fig. 4b).

**Fig. 5:**
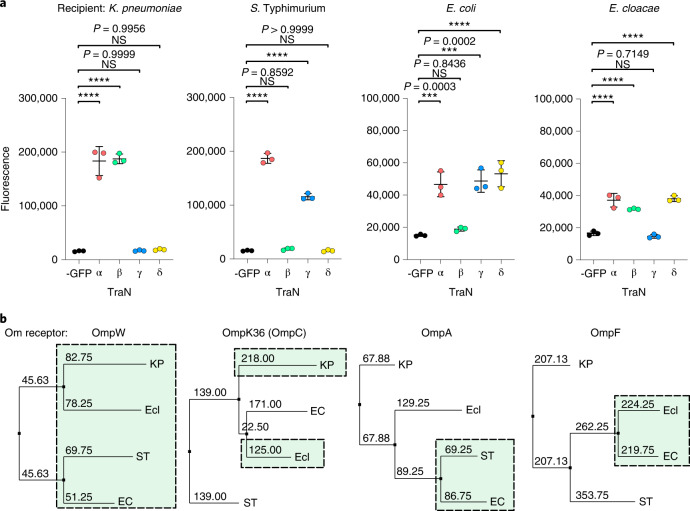
TraN structural variants mediate conjugation species specifity. **a**, RTCS endpoint measurements were taken from conjugation mixtures containing donors expressing different TraN tip structural variants and WT *K. pneumoniae*, *S*. Typhimurium, *E. coli* and *E. cloacae* recipients. A negative control was included for each recipient using a donor carrying the derepressed but untagged pKpQIL (-GFP). Fluorescence readings are presented as mean ± s.d. of three biological repeats analysed by one-way ANOVA with Dunnett’s multiple comparison test comparing to the negative control. ****P < 0.0001. **b**, Phylogenetic tree of TraN receptors from the four bacterial species used as recipients in conjugation assays: KP*, K. pneumoniae*; EC, *E. coli*; ST*, S*. Typhimurium; Ecl, *E. cloacae*. Homologues that participate in MPS with a corresponding TraN are highlighted in the dashed boxes. Source data

Conjugation facilitated by representative TraNs from each group into WT *K. pneumoniae*, *E. coli*, *S*. Typhimurium and *E. cloacae* recipients was assessed using RTCS (Fig. 5a). In alignment with the plasmid host distribution, TraNα mediated efficient transfer into all recipients tested, while TraNβ and TraNγ were associated with species-specific conjugation. In contrast, while plasmids with TraNδ showed a broad host range distribution, species-specific transfer was observed in vitro. This suggests that MPS-mediated conjugative transfer influences host distribution, although this effect may not be as apparent where the sample size of plasmids is small (that is, TraNδ). Phylogenetic analysis of the OM protein receptors from each species revealed clustering of homologues which appear to interact with TraN on the basis of the functional assays, except for homologues of OmpK36 which interact with TraNβ (Fig. 5b).

## Discussion

Although TraN has previously been implicated in MPS, we have now elucidated the mechanism by which it mediates intimate contacts between conjugating bacteria. To the best of our knowledge, this is presumably the first example of OM proteins in opposing cells cooperating to mediate bacterial–bacterial interactions.

**Fig. 6:**
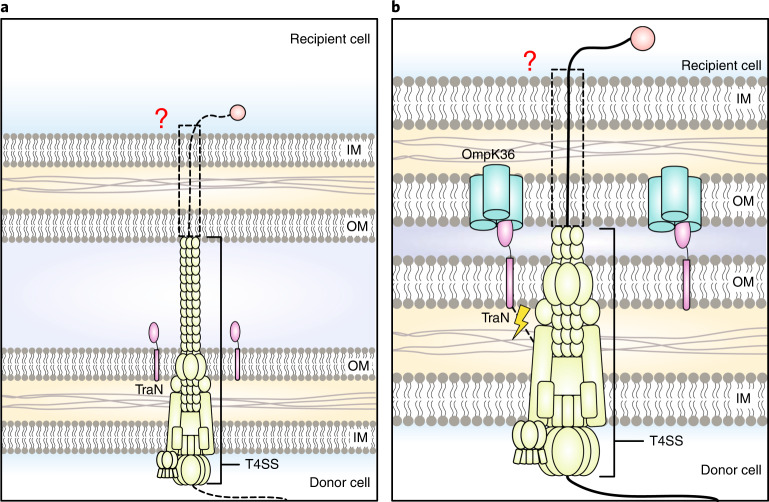
Proposed model of conjugation. **a**, Low-efficiency DNA transfer occurs in the absence of MPS. Where a suitable TraN receptor is not present in the recipient, conjugation occurs without intimate attachment, resulting in a baseline conjugation frequency. **b**, High-efficiency transfer occurs when MPS is successful, potentially through the intimate attachment of cells and the transmission of a signal to the core machinery. The conduit for DNA transfer across the recipient membranes remains unknown and is represented by the dashed box. IM, inner membrane.

While MPS facilitates efficient conjugation, low-frequency transfer occurs in the absence of a suitable receptor and can be increased following transfer gene derepression. On the basis of this, we propose a model in which the pilus mediates a baseline level of conjugation in the absence of MPS (Fig. 6a). This form of transfer is dependent on the expression level of donor transfer genes and is probably less efficient as cells are not intimately attached. Several observations of conjugation occurring in the absence of intimate cell–cell attachment indeed support the idea that the pilus can serve as a conduit for relaxase-bound DNA entry into the recipient^36,37^. Where recipients expressing the appropriate OMPs are present, low-efficiency transfer is supplemented by high-efficiency transfer mediated by MPS following pilus retraction. In addition to ensuring that mating pairs stay intimately attached throughout DNA transfer, the interaction of TraN with a suitable receptor may also provide a signal to the core machinery that increases transfer efficiency (Fig. 6b). Our model builds upon the ‘shoot and pump’ model first proposed by Llosa et al.^38^, where recipients cannot avoid conjugation. Ultimately, conjugation will always occur where there is the expression of functional transfer machinery to impart the sheer push force required to deliver DNA into the recipient cell, while MPS serves to improve the efficiency of this process.

Analysis of TraN sequences found on fully sequenced plasmids revealed a clear correlation of several homologues with a single dominant species, the molecular basis of which is reflected in our in vitro findings of TraN pairing with specific OM proteins in the recipient. One feature of the dataset that should be acknowledged is the bias towards bacterial species that are highly associated with antimicrobial resistance and nosocomial infections in developed countries. Therefore, while our analysis may be useful in highlighting TraN variants that facilitate conjugation of high-risk plasmids in pathogens of interest, it may not reflect the entire range of receptive host species. Some epidemic plasmids associated with resistance or virulence may also be overrepresented. Of note, pKpQIL-like plasmids are found extensively within clinical ST258 isolates^39^. However, these plasmids have also been detected in other sequence types of *K. pneumoniae* and other bacterial species, suggesting that conjugation still plays a prominent role in its dissemination^20,39^.

In summary, our findings highlight the importance of TraN-mediated MPS in driving high-efficiency transfer of IncF plasmids and its role in shaping plasmid host range. Our findings suggest that targeted strategies could be developed to interrupt MPS for high-risk IncF plasmids. Notably, despite great diversity in TraN sequence variants, many are structurally conserved and, therefore, mediate conjugation via the same receptors. Accordingly, we have now classified approximately 90% of the plasmids in our curated dataset into one of four groups (TraNα–δ) on the basis of the TraN structural variant expressed and suggest that conjugation-blocking strategies be prioritized for these four known structures. We envision that a combination of *traN* sequencing with rapid structure prediction could form the basis of future surveillance strategies for emergent high-risk plasmids as a means of predicting dissemination into important pathogens.

## Methods

### Bacterial strains and plasmids

The bacterial strains, conjugative IncF plasmids and mutagenesis vectors used are listed in Supplementary Tables 1, 3 and 4, respectively. Unless otherwise stated, bacteria were cultured in Lysogeny Broth (LB) at 37 °C, 200 r.p.m. When needed, antibiotics were used at the following concentrations: ertapenem (0.5 μg ml^−1^), streptomycin (50 μg ml^−1^), kanamycin (50 μg ml^−1^), gentamicin (10 μg ml^−1^).

### Generation of mutants

All genomic mutations were made in ICC8001, a rifampicin-resistant derivative of *K. pneumoniae* ATCC 43816 using a two-step recombination methodology. Mutagenesis vectors were mobilized from *E. coli* CC118λpir into pACBSR-carrying strains through a tri-parental conjugation using the *E. coli* 1047 pRK2013 helper strain. Merodiploid colonies were selected on LB agar containing gentamicin and streptomycin. Selected colonies were grown for at least 4 h in LB supplemented with streptomycin and 0.4% l-arabinose to induce expression of the I-SceI endonuclease from the pACBSR plasmid. Cultures were streaked onto LB agar containing streptomycin and screened for the intended mutations. Mutations in pKpQIL were introduced using the same methodology.

Mutagenesis vectors were generated by Gibson Assembly (New England Biolabs, E2611L) on the pSEVA612S backbone and were maintained in CC118λpir cells. Site-directed mutagenesis on previously generated vectors was performed according to the Q5 Site-Directed Mutagenesis Kit protocol (New England Biolabs, M0554S). Primers used to generate the mutagenesis vectors and for screening are listed in Supplementary Table 5. All mutations were confirmed by sequencing (Eurofins). The R100-1 plasmid was provided by Fernando de la Cruz and transformed into chemically competent DH5α, which served as the donor strain in conjugation assays. GeneArt Gene Synthesis (ThermoFisher) was used to synthesize a nucleotide string encoding the tip region of TraN_MV1_.

### Selection-based conjugation assays

For experiments using donors carrying pKpGFP and its derivatives, recipients were transformed with pSEVA471, a low-copy-number plasmid encoding streptomycin resistance. For quantification of R100-1 conjugation, DH5α carrying R100-1 was used as the donor and recipients were transformed with pUltra-sfGFP, which confers gentamicin resistance. For all experiments, overnight cultures of donor and recipient bacteria were washed in phosphate-buffered saline (PBS). Donor and recipient cells were mixed at a ratio of 8:1, which was previously determined to result in the highest conjugation frequency for pKpQIL^40^, and diluted in PBS (1 in 25 v/v). A volume of 40 μl of the final conjugation mixture was spotted onto LB agar and incubated for 6 h at 37 °C. The spots were collected and resuspended in 1 ml of sterile PBS for serial dilution. Recipient colonies were selected on streptomycin- or gentamicin-containing LB agar plates. Transconjugants were selected on plates supplemented with streptomycin and ertapenem for pKpGFP experiments, and streptomycin and gentamicin for R100-1 conjugation experiments. Plates were visualized on a Safe Imager 2.0 Blue Light Transilluminator (ThermoFisher) to confirm plasmid uptake in transconjugant colonies by GFP fluorescence. Conjugation frequency was calculated as the ratio of the colony forming units (c.f.u.) per ml of transconjugants to the c.f.u. per ml of recipients and the data were log_10_ transformed before statistical analysis.

### RTCS assays

Conjugation mixtures were prepared by mixing PBS-washed overnight cultures of donors carrying derepressed reporter plasmids and recipient bacteria. It was determined that maximal fluorescence emission was obtained when donor and recipient bacteria were mixed at a 1:1 ratio without dilution. The conjugation mixture (8 µl) was spotted onto 270 µl LB agar in a 96-well black microtitre plate in technical triplicate. The plates were incubated for 6 h at 37 °C, with fluorescence readings taken at 10 min intervals on a FLUOstar Omega (BMG Labtech). Fluorescence data at each timepoint were calculated by normalizing the raw GFP emission at that timepoint to the minimum GFP emission recorded for each sample over the 6 h time course. Arbitrary fluorescence units (a.f.u.) were determined by calculating the log fold change of fluorescence at *t* = 300 min for each mutant recipient strain (X) against the WT recipient, that is, a.f.u. = log_10_(fluorescence_X_/fluorescence_WT_).

### Purification of conjugative pili and generation of anti-pili antibodies

GFP-DD overnight cultures (2 l) were collected by centrifugation at 7,000 × *g* for 20 min and resuspended in 40 ml of cold 1X PBS. The resuspended cells were passed through a 25 G needle 30 times. ‘Shaved’ bacteria were centrifuged at 50,000 × *g* for 1 h. The supernatant was mixed with 5% PEG 6000, with constant stirring for 1 h at 4 °C. Conjugative pili were precipitated by centrifugation at 50,000 × *g* for 30 min. The pellet was resuspended in a buffer containing 50 mM Tris pH8, 1 M NaCl and dialysed overnight against the same buffer. The purified pili were visualized by negative stain electron microscopy to assess for pilus integrity and purity. Rat polyclonal antibodies were raised against the purified pili (ThermoFisher). Polyclonal antibodies were adsorbed against paraformaldehyde-fixed wild type *K. pneumoniae* to isolate antibodies specific to the conjugative pilus.

### Immunofluorescence microscopy

Overnight cultures were diluted 1 in 20 (v/v) in fresh LB and 300 ml was added to glass coverslips placed in a 24-well plate before incubation at 37 °C for 1.5 h to allow bacteria to adhere to the surface of the coverslips. Excess medium was removed and the coverslips were washed with PBS before fixation in 4% paraformaldehyde for 20 min at room temperature. Fixed samples were washed in PBS and blocked in 2% bovine serum albumin (BSA) in PBS (w/v). Samples were washed three times before incubation with anti-pili antibodies (1:100 in 2% BSA/PBS) for 1 h at room temperature. Samples were washed three times in PBS and incubated with Alexa Fluor 488 conjugated Donkey anti-rat IgG antibodies (Jackson Immunoresearch, 712-546-150; 1:1,000 in 2% BSA/PBS) for 1 h at room temperature. Coverslips were washed three times in PBS and incubated with FM4-64 (Invitrogen, T13320; 1:100 in water) for 5 min at room temperature. Following this, coverslips were dried and mounted onto glass slides using VECTASHIELD Hardset Antifade mounting medium with DAPI (Vector Laboratories, H-1500-10) according to the manufacturer’s instruction. Slides were analysed using a ×100 objective lens on a Zeiss Axio Observer 7 microscope and images were processed on Zen 2.3 (Blue Version, Zeiss). Images shown are representative of at least three biological repeats.

### Agarose pad live microscopy

Bacterial conjugation was visualized over time on a Celldiscoverer 7 live cell imaging microscope (Zeiss). For these experiments, the GFP-DD donor strain was mixed with 36_WT_-expressing recipients that constitutively express dTomato. Overnight cultures of donor and recipient bacteria were washed in PBS, mixed in a 1:1 ratio and 8 μl was spotted onto a 1 cm^2^ 2% agarose (w/v) pad supplemented with M9 salts and 0.4% glucose (w/v). The pad was inverted into a μ-Slide 2-well chambered coverslip (Ibidi, 80286). The sample was maintained at 37 °C throughout live imaging. Images were acquired every 10 min for 3.5 h and processed using Zen 2.3 (Blue Version, Zeiss).

### Generation of TraN AlphaFold models

In the absence of homologous TraN structures, ab initio models were generated by AlphaFold v2.0^33^. TraN sequences were submitted to the AlphaFold Colab server with the default settings; the signal peptide was removed from all sequences before modelling. Each structural model was validated by analysing the confidence score as generated by the pLDDT. Molecular graphics and superimposition analysis were performed in UCSF ChimeraΧ-1.2.5^41^.

### Overexpression and purification of TraN and OmpK36

Recombinant OmpK36 was extracted from *E. coli* outer membranes using 1% *n*-Dodecyl-*N*,*N*-Dimethylamine-*N*-Oxide (LDAO; Anatrace) and was further purified by affinity chromatography and anion exchange^29^. The final OmpK36 buffer was 50 mM NaCl, 10 mM HEPES pH 7.0 and 0.03% *n*-dodecyl-β-d-maltoside (DDM; Anatrace). The mature TraN gene (D28 to Q651) from pKpQIL was subcloned into the pTAMAHISTEV vector with an N-terminal His_7_-tag and a tobacco etch virus (TEV) cleavage site using the NcoI and XhoI restriction enzyme sites. The construct was transformed into *E. coli* C43 (DE3) competent cells (F^−^ *ompT hsdS*_*B*_ (*r*_*B*_^*−*^ *m*_*B*_ −) *gal dcm* (DE3))^42^ and expressed in Terrific Broth (TB) medium (Formedium) supplemented with 100 µg ml^−1^ ampicillin. Cultures were grown to an OD_600_ of 0.6–0.8 at 37 °C, then induced with 0.5 mM isopropyl β-d-1-thiogalactopyranoside (IPTG) and maintained for 16 h at 25 °C. Outer membranes were produced as previously described^43^, then solubilized overnight in 1% DDM in 1X PBS. Insoluble material was pelleted at 131,000 × *g* for 1 h, and the supernatant was supplemented with 30 mM imidazole and loaded onto an Econo-Column (Biorad) containing 5 ml Ni-NTA resin (Qiagen). The column was washed with 5 column volumes of wash buffer (1X PBS, 30 mM imidazole and 0.1% DDM). TraN-His_7_ eluted from the resin in wash buffer containing 100–250 mM imidazole. TraN-His_7_ was dialysed against 50 mM NaCl, 10 mM HEPES pH 7.0 and 0.03% DDM (buffer A) while undergoing incubation with His_6_-tagged TEV protease for 16–18 h at 4 °C. The dialysed sample was passed over a 5 ml His-Trap column (Cytiva) and the cleaved protein was collected in the flowthrough. Fractions containing TraN were combined and further purified using anion-exchange chromatography (Mono Q 5/10 GL column; Cytiva) using an ÄKTA pure system (Cytiva). The column was equilibrated with buffer A and eluted using a linear gradient with 500 mM NaCl, 10 mM HEPES pH 7.0 and 0.03% DDM (buffer B) over 20 column volumes. TraN eluted in 18% buffer B and was concentrated to 1 mg ml^−1^ for SEC analysis.

### SEC analysis of TraN-OmpK36

TraN and OmpK36 were dialysed against buffer A (16–18 h at 4 °C) and then were combined at a 1:2 molar ratio respectively at 1 mg ml^−1^ and incubated for 16 h at room temperature. The sample was injected onto a Superose 6 10/300 GL column (Cytiva), equilibrated in buffer A and eluted at a flow rate of 0.3 ml min^−1^ while monitoring the absorbance at 280 nm. This was followed by separate injections of TraN and OmpK36 onto the column at the same molar concentrations as previously described for the comparison of retention volumes. Data were collected on UNICORN 7.5. Fractions were collected and analysed by SDS–PAGE.

### Cryo-EM sample preparation and data collection

Sample containing OmpK36-TraN at a concentration of 0.33 mg ml^−1^ was diluted 1:6 in buffer A. In brief, a 4 μl aliquot of sample was applied to a plasma-cleaned (Gatan Solarus) graphene oxide-coated Cu 300 mesh 1.2/1.3 holey carbon grid (Quantifoil), blotted with force 6 for 4.5 s at 90% humidity and flash frozen in liquid ethane using a Vitrobot Mark IV (FEI). The dataset used for structure determination was collected at the Molecular Electron Microscopy Core at the University of Virginia on a Titan Krios EM operated at 300 keV, equipped with an energy filter and K3 direct electron detector (Gatan). An energy filter slit width of 10 eV was used during data collection and was aligned automatically every hour. All 13,668 movies were collected in counting mode at a magnification of 81 K, pixel size of 1.08 Å, and a defocus range from −2.2 to −1.2 μm. Data collection was performed using a total dose of 50 e^−^ Å^−2^ across 40 frames at a rate of 4.78 s per movie.

### Data processing

Unless otherwise stated, all data processing was completed using cryoSPARC v3.2.0^44^. Movies were corrected for full-frame motion using Patch Motion Correction followed by Gctf contrast transfer function estimation. After contrast transfer function estimation, micrographs were sorted and selected on the basis of estimated resolution (better than 4 Å), defocus (−1 to −2.5 μm), ice thickness and total full-frame motion. Initial particles were automatically picked using ‘Blob picker’ with minimum and maximum particle diameters of 200 and 256 Å, respectively. Particles were extracted at a box size of 256 pixels, followed by two-dimensional (2D) classification. Class averages of trimeric OmpK36 alone and OmpK36 with TraN were selected for template-based particle picking. A total of 13,780,567 particles were extracted using a box diameter of 256 Å. These particles were sorted using 3 iterative rounds of 2D classification with 50 classes each, the number of online-EM iterations being set to 100 and the batch size to 1,000 per class. The final iteration of 2D classification yielded a subset of 3,412,946 particles.

To differentiate particles containing only OmpK36 or OmpK36 + TraN, multiple 3D maps were generated using ‘Ab initio reconstruction’, with class size set to 4. Output 3D maps were inspected for the presence of TraN. Particles were further refined using two iterations of heterogeneous refinement with input volumes created by multi-class ab initio. The highest resolution class from the second iteration of heterogeneous refinement contained 359,314 particles, which allowed for a ~2.6 Å map to be reconstructed using ‘non-uniform refinement’ (Extended Data Fig. 7 and Supplementary Table 6).

### Model building and refinement

The density for the trimeric OmpK36 allowed us to trace the entire backbone and build most side chains throughout the structure. The OmpK36 crystal structure (PDB ID: 6RD3)^29^ was used for building the cryo-EM model, which only had small differences relative to the starting model. The predicted TraN AlphaFold model was used for initial interpretation of the loop-shaped density found within the lumen of one porin channel. The loop and the two β-strands on either side of the hairpin of the AlphaFold model could be fit into the density. Two cysteines at either side of the hairpin fit into the TraN density and were used as a starting point for matching larger side chains within the density. Model building, including adjusting side chains, was performed in Coot^45^. The model was refined in Phenix v1.15.2-3472, using real-space refinement with ‘ignoring symmetry conflicts’ turned on^46^. Refinement included global minimization, B-factor optimization, and applied secondary structure and Ramachandran restraints. The final model had a MolProbity score of 1.39, with 96% and 0.1% in the Ramachandran favoured and outlier regions, respectively (Supplementary Table 6). The OmpK36-TraN complex coordinates have been deposited to the Protein Data Bank (https://www.rcsb.org/) with PDB ID 7SZI. The EM map has been submitted to Electron Microscopy Data Bank (https://www.ebi.ac.uk/pdbe/emdb/) with ID EMD 25567.

### Bioinformatic analysis of TraN variants

We analysed a previously described dataset of 14,029 sequenced plasmids deposited in GenBank for predicted conjugative plasmids using the Plascad tool for plasmid characterization^34^. Briefly, Plascad predicts and distinguishes conjugative plasmids from mobilizable and non-mobilizable plasmids on the basis of the presence of relaxase, T4CP and T4SS genes. It further characterizes conjugative plasmids into the four archetypal mating pair formation (MPF) groups. Putative MPF_F_ conjugative plasmids were extracted from the dataset. Next, the bacterial host family associated with each plasmid was determined by querying the NCBI Taxonomy database via the ‘ncbi_taxonomy’ module in the Python toolkit ‘ETE’ (v3.0) and manually curating the results. Finally, PlasmidFinder was used to define the plasmid replicons for the purpose of selecting plasmids carrying an IncF replicon. A curated dataset of 824 putative conjugative IncF plasmids found in an Enterobacteriaceae host was screened for *traN* variants (Supplementary Table 1). A tBLASTn^35^ was performed and required sequences to share ≥90% amino acid similarity with a reference and possess ≥75% of the reference length to categorize them as the same *traN* type. Phylogenetic trees were constructed with RAxML v8.2.8^47^ using the *traN* nucleotide sequences from plasmids carrying variants of the pKpQIL, R100-1 and F *traN* genes, midpoint-rooted and visualized with metadata using Microreact^48^ (version TBC). Further verification of annotated *traN* sequences was performed by analysing open reading frames in the reference plasmids for cysteine residue abundance on the basis that TraN is known to contain at least 20 cysteine residues (Supplementary Table 8).

### Multiple sequence alignments of TraN and OM proteins

Multiple sequence alignments were generated in Clustal Omega 1.2.4. TraN sequences were obtained from the following reference plasmids: pKpQIL (accession ID: KY798507.1), R100-1 (accession ID: DQ364638.1), F (accession ID: NC_002483.1), pSLT (accession ID: AE006471.2), MV1 (accession ID: NZ_CP016763.1), MV2 (accession ID: AP014954.1), MV3 (accession ID: NZ_CP023348.1). OM protein sequences were obtained from the following published genomes: *K. pneumoniae* ATCC 43816 (accession ID: CP009208), *E. coli* MG1655 (accession ID: U00096.3), *S*. Typhimurium LT2 (accession ID: AE006468), *E. cloacae* ATCC 13047 (accession ID: CP001918). Phylogenetic trees were calculated using the neighbour joining method and visualized in Jalview 2.11.2.2.

### Statistics and reproducibility

All data are representative of at least three biological repeats. All attempts at replication were successful. Statistical analyses were performed on Prism 9 (GraphPad software). Conjugation data were analysed by repeated measures one-way analysis of variance (ANOVA) with Tukey’s or Dunnett’s multiple comparison test, as appropriate. Where only two recipient strains were being compared, a two-sided paired *t*-test was used. A multiple *t*-test with Holm-Šídák correction was used to assess the effect of OmpK36 mutations in the donor. *P* values less than 0.05 were considered significant.

### Reporting summary

Further information on research design is available in the Nature Research Reporting Summary linked to this article.

## Supplementary information

Supplementary Information Supplementary Tables 1, 3–7.

Reporting Summary

Supplementary Table 1 Supplementary Table 2. List of IncF conjugative plasmids. Plasmids were grouped and coloured according to *traN* variant. The percentage identity of each *traN* to the reference *traN* as determined by tBLASTn is listed. Supplementary Table 8. Cysteine residues in plasmid ORFs. The table lists all annotated open reading frames in plasmids containing the reference *traN* genes and the corresponding number of cysteine residues encoded.

Supplementary Video 1 **Live microscopy of pKpGFP-D conjugation**. Donor cells carrying pKpGFP-D (GFP-DD) were mixed at a 1:1 ratio with WT recipients expressing dTomato (red) and observed by live microscopy. Transconjugant cells that have acquired the plasmid appear green. Each frame was captured 10 min apart. The video plays at ×2,400 actual speed.

## Source data

Source Data Fig. 1 Statistical source data.

Source Data Fig. 2 Statistical source data.

Source Data Fig. 5 Statistical source data.

Source Data Extended Data Fig. 1 Statistical source data.

Source Data Extended Data Fig. 2 Statistical source data.

Source Data Extended Data Fig. 3 Statistical source data.

Source Data Extended Data Fig. 4 Statistical source data.

Source Data Extended Data Fig. 6 Statistical source data.

Source Data Extended Data Fig. 6 Unprocessed Coomassie-stained SDS–PAGE gel.

Source Data Extended Data Fig. 10 Statistical source data.

## Data Availability

The data supporting the findings in this study are provided within the Article and its Supplementary Information. Accession IDs of published sequences for reference plasmids and genomes are listed in the Methods. The coordinates and structure factors of the TraN-OmpK36 complex have been deposited to the Protein Data Bank and Electron Microscopy Data Bank with ID codes 7SZI and 25567, respectively. Source data are provided with this paper.
