# Supplementary material for: Mating pair stabilization mediates bacterial conjugation species specificity
Source: Nat Microbiol. 2022 Jun 13;7(7):1016–27. doi: 10.1038/s41564-022-01146-4 (PMC9246713; doi:10.1038/s41564-022-01146-4)
Supplement: Supplementary file 1 — Supplementary Tables 1, 3–7. [file 41564_2022_1146_MOESM1_ESM.pdf]

nature portfolio

https://doi.org/10.1038/s41564-022-01146-4

Supplementary information

Mating pair stabilization mediates bacterial conjugation species specificity

In the format provided by the authors and unedited

Supplementary Information

Mating pair stabilisation mediates bacterial conjugation species specificity

Wen Wen Low[1,2], Joshua L.C. Wong[1,2^], Leticia C. Beltran[3^], Chloe Seddon[1,2,4^], Sophia David[5^], Hok-Sau Kwong[2,3], Tatiana Bizeau[2], Fengbin Wang[3], Alejandro Peña[1,2], Tiago R. D. Costa[1,2], Bach Pham[6], Min Chen[6], Edward H. Egelman[3], Konstantinos Beis[2,4*], Gad Frankel[1,2*]

[1]MRC Centre for Molecular Microbiology and Infection, Imperial College, London, UK

[2]Department of Life Sciences, Imperial College, London, UK

[3]Department of Biochemistry and Molecular Genetics, University of Virginia, Charlottesville, VA 22903, USA

[4]Rutherford Appleton Laboratory, Research Complex at Harwell, Didcot, Oxfordshire, UK

[5]Centre for Genomic Pathogen Surveillance, Big Data Institute, Li Ka Shing Centre for Health Information and Discovery, University of Oxford, Oxford, UK

[6]Department of Chemistry, University of Massachusetts Amherst Amherst, MA 01003, USA

[^]These authors contributed equally

[*]These authors jointly supervised this work

Corresponding author: Gad Frankel (g.frankel@imperial.ac.uk)

Table of Contents

Supplementary Table 1. Bacterial strains used in this study. 3

Supplementary Table 2.xlsx. List of IncF conjugative plasmids. 4

Supplementary Table 3. Conjugative IncF plasmids used and generated in this study. 5

Supplementary Table 4. Plasmids and vectors used for mutagenesis. 6

Supplementary Table 5. Primers used in this work. 7

Supplementary Table 6. Cryo-EM and Refinement Statistics of OmpK36-TraN complex. 10

Supplementary Table 7. Cysteine residues in predicted disulphide bonds in TraN. 11

Supplementary Table 8.xlsx. Cysteine residues in plasmid ORFs. 12

Supplementary Video. Live microscopy of pKpGFP-D conjugation. 12

Supplementary Table 1. Bacterial strains used in this study.

Strain	Description	Source
Cloning strains		
CC118λpir	Expresses the Pi protein for the replication of plasmids with the R6K origin.	Lab collection
E. coli 1047 pRK2013	Triparental conjugation helper strain. Kanamycin resistant	Lab collection
Klebsiella pneumoniae strains		
ICC8001	Parental wild type (WT) strain of *K. pneumoniae* ATCC43816 serially passaged *in vitro* on Rifampicin (100 µg/ml) followed by two passages in BALB/c mice.	Lab collection
Donor strains		
ICC8005	Donor strain for conjugation assays. Tagged with constitutive Biofab promoter-driven *lacI* construct at 3' end of *glmS* gene	This study
GFP-D	ICC8005 carrying pKpGFP	This study
GFP-DD	ICC8005 carrying pKpGFP-D	This study
GFP-DΔ*traN*	ICC8005 carrying pKpGFPΔ*traN*	This study
GFP-DDΔ*traN*	ICC8005 carrying pKpGFP-DΔ*traN*	This study
GFP-D*traN*$_{R100-1}$	ICC8005 carrying pKpGFP*traN*$_{R100-1}$	This study
GFP-DD*traN*$_{R100-1}$	ICC8005 carrying pKpGFP-D*traN*$_{R100-1}$	This study
GFP-DD*traN*$_{Ch1}$	ICC8005 carrying pKpGFP*traN*$_{Ch1}$. Tip region of TraN corresponds to TraN$_{R100-1}$.	This study
GFP-DtraN$_{F}$	ICC8005 carrying pKpGFP*traN*$_{F}$	This study
GFP-DD*traN*$_{F}$	ICC8005 carrying pKpGFP-D*traN*$_{F}$	This study
GFP-DD*traN*$_{Ch2}$	ICC8005 carrying pKpGFP*traN*$_{Ch2}$. Tip region of TraN corresponds to TraN$_{pSLT}$.	This study
GFP-DD*traN*$_{Ch3}$	ICC8005 carrying pKpGFP*traN*$_{Ch3}$. Tip region of TraN corresponds to TraN$_{MV1}$.	This study
Recipient strains		
Δ35	ICC8001 Δ*ompK35*	Lab collection
Δ35/36$_{ST258}$	ICC8001 Δ*ompK35* expressing ST258 variant of *ompK36*	Lab collection
Δ35/Δ36	ICC8001 Δ*ompK35*Δ*ompK36*	Lab collection
Δ35/36$_{ST258\Delta GD}$	ICC8001 Δ*ompK35*, *ompK36*$_{ST258}$ with L3 GD deletion	Lab collection
Δ35/36$_{WT+GD}$	ICC8001 Δ*ompK35*, *ompK36*$_{WT}$ with L3 GD insertion	Lab collection
ICC8006	Recipient strain used for live microscopy experiment. Tagged with Biofab-promoter driven *dTomato* construct at 3' end of *glmS* site	This study
ICC8007	ICC8001 Δ*ompA*	This study
ICC8008	ICC8001 Δ*ompW*	This study
ICC8009	ICC8001 Δ*phoE*	This study
Escherichia coli strains		
DH5α R100-1	*E. coli* donor for R100-1	This study
MG1655	*E. coli* K-12 strain	Lab collection
ICC8010	MG1655 Δ*ompA*	This study
ICC8011	MG1655 Δ*ompF*	This study
ICC8012	MG1655 Δ*ompC*	This study
Others		
SV3081	*Salmonella enterica* serovar Typhimurium LT2 recipient strain. Cured of pSLT	Gift from Josep Casadesus
ATCC 13047	*Enterobacter cloacae* recipient strain.	Gift from Avinash Shenoy

Supplementary Table 2.xlsx. List of IncF conjugative plasmids.

Plasmids were grouped and colored according to *traN* variant. The percentage identity of each *traN* to the reference *traN* as determined by tBLASTn is listed.

Supplementary Table 3. Conjugative IncF plasmids used and generated in this study.

Plasmid	Description		Source
pKpQIL	IncFII$_{K}$ *bla*$_{KPC-2}$-encoding plasmid. Shares sequence with pKpQIL-UK (Genbank accession no. KY798507)		Lab collection
R100-1	Derepressed variant of R100 (IncFII)		Provided by Fernando de la Cruz
pOX38	F plasmid derivative		Lab collection
pSLT	IncFII$_{S}$ *S.* Typhimurium virulence plasmid		Lab collection
Synthetic reporter plasmids			
**ID**	**Plasmid**	**Description**	**Source**
pICC4000	pKpGFP	pKpQIL with P*lac-sfGFP* construct inserted at disrupted *aadA* gene. Parental reporter plasmid.	This study
pICC4003	pKpGFP-D	pICC4000Δ*finO.* Derepressed reporter plasmid	This study
pICC4005	pKpGFP*traN*$_{R100-1}$	pICC4000Δ*traN*::*traN*$_{R100-1}$	This study
pICC4006	pKpGFP-D*traN*$_{R100-1}$	pICC4000Δ*finO*Δ*traN*::*traN*$_{R100-1}$	This study
pICC4007	pKpGFP-D*traN*$_{Ch1}$	pICC4000Δ*finO*Δ*traN*::*traN*$_{Ch1}$	This study
pICC4008	pKpGFP*traN*$_{F}$	pICC4000Δ*traN*::*traN*$_{F}$	This study
pICC4009	pKpGFP-D*traN*$_{F}$	pICC4000Δ*finO*Δ*traN*::*traN*$_{F}$	This study
pICC4010	pKpGFP-D*traN*$_{Ch2}$	pICC4000ΔfinOΔtraN::traN$_{Ch2}$	This study
pICC4011	pKpGFP-D*traN*$_{Ch3}$	pICC4000ΔfinOΔtraN::traN$_{Ch3}$	This study
pICC4002	pKpQILΔ*finO*	Derepressed pKpQIL. No P*lac-sfGFP* construct.	This study

Supplementary Table 4. Plasmids and vectors used for mutagenesis.

Vector	Description	Source
pACBSR	SmR; expresses I-SceI and lambda-red induced by L-Ara.	Lab collection
pSEVA612S	GmR; integrative plasmid (ori R6K) that harbours the oriT for conjugation.	Lab collection
pSEVA471	SmR; for selecting recipient cells in conjugation assays	Lab collection
pSEVA612S-Kp-N2-sfGFP	pSEVA612S derivative; inserts Biofab promoter driven *sfGFP* construct at 3' end of *glmS*	Lab collection
pET-28a(+)	KanR; used as template to amplify *lacI* gene	Lab collection
pCSCMV-dTomato	Used as template to amplify *dTomato*	Lab collection
pSEVA612SbiofablacI	pSEVA612S derivative; inserts Biofab promoter driven *lacI* construct at 3' end of *glmS*	This study
pSEVA612SPlacsfGFP	pSEVA612S derivative; inserts *lac* promoter driven *sfGFP* at disrupted *aadA* gene on pKpQIL	This study
pSEVA612SΔfinO	pSEVA612S derivative, deletes *finO* from pKpQIL	This study
pSEVA612SΔtraN	pSEVA612S derivative, deletes *traN* from pKpQIL	This study
pSEVA612Sdtomato	pSEVA612S derivative; inserts Biofab promoter driven *dTomato* construct at 3' end of *glmS*	This study
pSEVA612StraNR100	pSEVA612S derivative; substitutes the ORF of *traN* in pKpQIL with the ORF for *traN* from R100-1	This study
pSEVA612StraNCh1	pSEVA612S derivative; substitutes the ORF of *traN* in pKpQIL with a chimeric *traN* expressing tip of $\text{TraN}_{\text{R100-1}}$	This study
pSEVA612SΔompAKP	pSEVA612S derivative; deletes *ompA* from *K. pneumoniae*	This study
pSEVA612SΔompW	pSEVA612S derivative; deletes *ompW*	This study
pSEVA612SΔphoE	pSEVA612S derivative; deletes *phoE*	This study
pSEVA612StraNF	pSEVA612S derivative; substitutes the ORF of *traN* in pKpQIL with the ORF for *traN* from F	This study
pSEVA612SΔompAEC	pSEVA612S derivative; deletes *ompA* from *E. coli*	This study
pSEVA612StraNCh2	pSEVA612S derivative; substitutes the ORF of *traN* in pKpQIL with a chimeric *traN* expressing tip of $\text{TraN}_{\text{pSLT}}$	This study
pSEVA612StraNCh3	pSEVA612S derivative; substitutes the ORF of *traN* in pKpQIL with a chimeric *traN* expressing tip of TraN_{MV1}	This study
pSEVA612SΔompF	pSEVA612S derivative; deletes *ompF* from *E. coli*	This study
pSEVA612SΔompC	pSEVA612S derivative; deletes *ompC* from *E. coli*	This study

Supplementary Table 5. Primers used in this work.

Primer	Sequence (5' - 3')	Description
pSEVA612S_F	ATTACCCTGTTATCCCTATACTG	Amplifies linear pSEVA612S
pSEVA612S_R	TAGGGATAACAGGGTAATCCG	
lacIvector_F	TAAGGATCCAACAGGGTTC	Amplifies linear pSEVA612S with homology regions flanking the 3' end of *glmS* and Biofab promoter from pSEVA612S-Kp-N2-sfGFP
lacIvector_R	TTTTTTTTTACCTCCTTAAACTCC	
lacI_F	TTTAAGGAGGTAAAAAAAAAGTGGTGAATGTGAAACCAGTAAC	Amplifies *lacI*
lacI_R	AGAACCCTGTTGGATCCTTATCACTGCCCGCTTTCCAG	
glmS_F	GGTCAGGATGCGTCTATCG	Checks for insertion at 5' end of *glmS*
glmS_R	CCTGAGTCAGTTTGTCATC	
aadAUPHR_F	GGATTACCCTGTTATCCCTACAAACGCGAAGGCCGGTG	Amplifies upstream homology region flanking disrupted *aadA* on pKpQIL
aadAUPHR_R	TTTTCTCGACGCGCGAGGCCAAGCGATC	
aadADNHR_F	GTACAAATAAGCAGATCAGTTGGAAGAATTTG	Amplifies downstream homology region flanking disrupted *aadA* on pKpQIL
aadADNHR_R	TATAGGGATAACAGGGTAATGCAAGATTCCACTATCAAAC	
plac_F	GGCCTCGCGCGTCGAGAAAATTTATCAAAAAGAGTG	Amplifies lac promoter
plac_R	CTTTACGCATACGTATCCTCCAAGCCTG	
sfGFP_F	GAGGATACGTATGCGTAAAGGCGAAGAG	Amplifies *sfGFP*
sfGFP_R	ACTGATCTGCTTATTTGTACAGTTCATCCATACC	
finOUPHR_F	GGATTACCCTGTTATCCCTACCCGTGGTATTCCGGGAATATTC	Amplifies upstream homology region of *finO*
finOUPHR_R	GTAAATATAAAACAATTGCCTATCGTTCAGTTAATAAG	
finODNHR_F	GGCAATTGTTTTATATTTACCCATTCCTGATAATTATACCTGGG	Amplifies downtream homology region of *finO*
finODNHR_R	TATAGGGATAACAGGGTAATCGGCAACATCGTCTCCCC	
finOext_F	GTTCTATGCTGTGCACCTGG	Checks for deletion of *finO*
finOext_R	GTTATGATGCCGCAGCCTG	
traNUPHR_F	GGATTACCCTGTTATCCCTACCGCCAGTTTATCGATAATCTG	Amplifies upstream homology region of *traN* from pKpQIL
traNUPHR_R	GCAGCATGGTTTCTGCCCTCCCTCATCC	
traNDNHR_F	GAGGGCAGAAACCATGCTGCCTAATAAAGAG	Amplifies downtream homology region of *traN* from pKpQIL
traNDNHR_R	TATAGGGATAACAGGGTAATGGAATAGCGGCATGCTCAG	
traNext_F	GGAGAAAGTGGCACAAACCG	Checks for deletion or substitution of *traN* on pKpQIL
traNext_R	CTTCCCGACGTCCCTTTGAC	
dTomato_F	TTTAAGGAGGTAAAAAAAAAATGGTGAGCAAGGGCGAG	Amplifies *dTomato*
dTomato_R	AGAACCCTGTTGGATCCTTATTACTTGTACAGCTCGTCCATG	

traNvector_F	AGGACAGTAAACCATGCTGCCTAATAAAGAG	Amplifies linear pSEVA612S with homology regions flanking *traN* from pKpQIL
traNvector_R	TACGTTTCATTTCTGCCCTCCCTCATCC	
traNR100_F	GAGGGCAGAAATGAAACGTATTTTACCTCTG	Amplifies *traN* from R100-1
traNR100_R	GCAGCATGGTTTACTGTCCTGCCTGTTTC	
KPCtraN_F	AGGGATGAGGGAGGGCAGAAATGAAGACGGTTATTTCCG	Amplifies *traN* from pKpQIL
KPCtraN_R	TCTTTATTAGGCAGCATGGTTTATTGCGCGGATTGCTG	
KPC169vector_F	ACCCTGGTGATGGAAGAAAC	Amplifies linear pSEVA612S with portion of *traN* from pKpQIL flanking the variable region
KPC169vector_R	CCGTGTGCAAAAATTTTCC	
traNR100-160_F	TGGAAAAATTTTTGCACACGGACTGCCAGTATCACCGGG	Amplifies variable region from *traN* of R100-1
traNR100-160_R	GTTTCTTCCATCACCAGGGTCAGCGTAAAGGTGGAAGC	
ompAUPHR_F	GGATTACCCTGTTATCCCTAGGAGTTAACCGCTGACGAAC	Amplifies upstream homology region flanking *ompA* from *K. pneumoniae*
ompAUPHR_R	CGGTTATAACTTTTTGCGCCTCATTATCATCC	
ompADNHR_F	GGCGCAAAAAGTTATAACCGATAAAAAAACCCGCTTC	Amplifies downstream homology region flanking *ompA* from *K. pneumoniae*
ompADNHR_R	TATAGGGATAACAGGGTAATCCCGCTACATTGAGGCCAG	
ompAext_F	CTTACGCTGCATGTATCAG	Checks for deletion of *ompA* in *K. pneumoniae*
ompAext_R	CAGGTAGGATCGTCGAC	
ompWUPHR_F	GGATTACCCTGTTATCCCTACCGGTTTTCATAAATAGTGC	Amplifies upstream homology region flanking *ompW*
ompWUPHR_R	ACAGAAGAATATCCACTTCCTCATTATGG	
ompWDNHR_F	GGAAGTGGATATTCTTCTGTAAACTGGCCAACG	Amplifies downstream homology region flanking *ompW*
ompWDNHR_R	TATAGGGATAACAGGGTAATGGCCAGGGGAGACCTATG	
ompWext_F	CTGAGGACTTAGTGTGATC	Checks for deletion of *ompW*
ompWext_R	CCCTGCTCAACATGTATCAC	
phoEUPHR_F	GGATTACCCTGTTATCCCTAAGGCGATGGTGGCGGGCA	Amplifies upstream homology region flanking *phoE*
phoEUPHR_R	CTGCGGTTAATATTCAGTCCTGGTGATTTATTTATACGCG CTATTCAATTGCG	
phoEDNHR_F	GGACTGAATATTAACCGCAGAACCACCC	Amplifies downstream homology region flanking *phoE*
phoEDNHR_R	TATAGGGATAACAGGGTAATATTGATAGCGGATCGGAC	
phoEext_F	CGGCGTTAAAAAACCTCC	Checks for deletion of *phoE*
phoEext_R	CTGCCGAAGGAGTATAAC	
traNF_F	GAGGGCAGAAATGAAACGTATTTTACCTCTG	Amplifies *traN* from F
traNF_R	GCAGCATGGTTTACTGTCCTGCCTGTTTC	
ompAECUPHR_F	GGATTACCCTGTTATCCCTAGACTGAAGAAGAGCATGC	

ompAECUPHR_R	AGACGAGAACTTTTTGCGCCTCGTTATC	Amplifies upstream homology region flanking *ompA* from *E. coli* MG1655
ompAECDNHR_F	GGCGCAAAAAGTTCTCGTCTGGTAGAAAAAC	Amplifies downstream homology region flanking *ompA* from *E. coli* MG1655
ompAECDNHR_R	TATAGGGATAACAGGGTAATGAAAGCGGTTGGAAATGG	
traNpSLT_F	TGGAAAATTTTTGCACACGGACGGCCACCATCACCGGC	Amplifies variable region from *traN* of pSLT
traNpSLT_R	GTTTCTTCCATCACCAGGGTCAGCGTGAACGTCGTTCTCCC	
traNMV1_F	TGGAAAATTTTTGCACACGGACGGCCAGCATTACCGGC	Amplifies variable region from *traN*$_{MV1}$
traNMV1_R	GTTTCTTCCATCACCAGGGTAACGGTGAAGCTGTAGCGC	
ompFUPHR_F	GGATTACCCTGTTATCCCTACGATCATCCTGTTACGGAATATTAC	Amplifies upstream homology region flanking *ompF* from *E. coli* MG1655
ompFUPHR_R	AGGTGTGCTATATTTATTACCCTCATGGTTTTTTTTATG	
ompFDNHR_F	GTAATAAATATAGCACACCTCTTTGTTAAATGCCGAAAAAACAGGACTTTG	Amplifies downstream homology region flanking *ompF* from *E. coli* MG1655
ompFDNHR_R	TATAGGGATAACAGGGTAATCGCCAGTGCCCCCCGGAG	
ompFext_F	GCAGACACATAAAGACAC	Checks for deletion of *ompF*
ompFext_R	GAGATTGCTCTGGAAG	
ompCUPHR_F	GGATTACCCTGTTATCCCTAGTGAAATAGTTAACAAGCG	Amplifies upstream homology region flanking *ompC* from *E. coli* MG1655
ompCUPHR_R	TCAATCGAGAGTTATTAACCCTCTGTTATATGC	
ompCDNHR_F	GGTTAATAACTCTCGATTGATATCGAACAAAGGGC	Amplifies downstream homology region flanking *ompC* from *E. coli* MG1655
ompCDNHR_R	TATAGGGATAACAGGGTAATGATTCACCAGCGGCCCGA	
ompCext_F	GTATCATATTCGTGTTGG	Checks for deletion of *ompC*
ompCext_R	GTACGCTGAAAACAATG	

Supplementary Table 6. Cryo-EM and Refinement Statistics of OmpK36-TraN complex.

**Parameter**	**OmpK36-TraN**
**Data collection and processing**	
Voltage (kV)	300
Electron exposure (e^{-} $Å^{-2}$)	50
Pixel size (Å)	1.08
Particle images (n)	359,314
Point group	C1
**Map resolution (Å)**	
Software (final reconstruction)	cryoSPARC
Model:map FSC (0.5)	2.7
Map:map FSC (0.143)	2.6
**Refinement and Model validation**	
Bond lengths rmsd (Å)	0.002
Bond angles rmsd (°)	0.441
Clashscore	3.61
Ramachandran Favored (%)	96.39
Ramachandran Outlier (%)	0.1
MolProbity score	1.39
**Deposition ID**	
PDB (model)	7SZI
EMDB (map)	EMD-25567

Supplementary Table 7. Cysteine residues in predicted disulphide bonds in TraN.

TraN_pKpQIL		TraN_R100-1		TraN_F	
CYS #1	CYS #2	CYS #1	CYS #2	CYS #1	CYS #2
114	417	114	392	114	378
129	372	129	347	129	333
139	319	139	294	139	280
203[a]	213[a]	244[a]	250[a]	294	316
242[a]	250[a]	308	330	342	367
333	355	356	381	349	358
381	406	363	372	384	389
388	397	398	403	435	446
423	428	449	460	445	460
474	485	459	474	483	491
484	499	497	505	499	530
522	530	513	544		
538	569				

[a]Cysteine residues in the tip domain of TraN

Supplementary Table 8.xlsx. Cysteine residues in plasmid ORFs.

The table lists all annotated open reading frames in plasmids containing the reference *traN* genes and the corresponding number of cysteine residues encoded.

Supplementary Video. Live microscopy of pKpGFP-D conjugation.

Donor cells carrying pKpGFP-D, (GFP-DD) were mixed at a 1:1 ratio with WT recipients expressing dTomato (red) and observed by live microscopy. Transconjugant cells which have acquired the plasmid appear green. Each frame was captured 10 min apart. The video plays at 2400X actual speed.
