# Supplementary material for: Mating pair stabilization mediates bacterial conjugation species specificity
Source: Nat Microbiol. 2022 Jun 13;7(7):1016–27. doi: 10.1038/s41564-022-01146-4 (PMC9246713; doi:10.1038/s41564-022-01146-4)
Supplement: Supplementary file 2 — Reporting Summary [file 41564_2022_1146_MOESM2_ESM.pdf]

nature portfolio

Corresponding author(s): Gad Frankel

Last updated by author(s): Apr 22, 2022

Reporting Summary

Nature Portfolio wishes to improve the reproducibility of the work that we publish. This form provides structure for consistency and transparency in reporting. For further information on Nature Portfolio policies, see our Editorial Policies and the Editorial Policy Checklist.

Statistics

For all statistical analyses, confirm that the following items are present in the figure legend, table legend, main text, or Methods section.

n/a	Confirmed	
☐	☒	The exact sample size (*n*) for each experimental group/condition, given as a discrete number and unit of measurement
☐	☒	A statement on whether measurements were taken from distinct samples or whether the same sample was measured repeatedly
☐	☒	The statistical test(s) used AND whether they are one- or two-sided<br>*Only common tests should be described solely by name; describe more complex techniques in the Methods section.*
☒	☐	A description of all covariates tested
☐	☒	A description of any assumptions or corrections, such as tests of normality and adjustment for multiple comparisons
☐	☒	A full description of the statistical parameters including central tendency (e.g. means) or other basic estimates (e.g. regression coefficient) AND variation (e.g. standard deviation) or associated estimates of uncertainty (e.g. confidence intervals)
☐	☒	For null hypothesis testing, the test statistic (e.g. *F*, *t*, *r*) with confidence intervals, effect sizes, degrees of freedom and *P* value noted<br>*Give P values as exact values whenever suitable.*
☒	☐	For Bayesian analysis, information on the choice of priors and Markov chain Monte Carlo settings
☒	☐	For hierarchical and complex designs, identification of the appropriate level for tests and full reporting of outcomes
☒	☐	Estimates of effect sizes (e.g. Cohen's *d*, Pearson's *r*), indicating how they were calculated

Our web collection on statistics for biologists contains articles on many of the points above.

Software and code

Policy information about availability of computer code

Data collection	RTCS data was collected using BMG Labtech OMEGA 5.70 and exported for further analysis via BMG Labtech MARS Data Analysis 3.42 R5.<br>Immunofluorescence microscopy data acquisition was collected using Zeiss Zen Pro 2.3.<br>SEC data was collected using UNICORN 7.5.<br>Ab initio models of TraN were generated in AlphaFold v2.0
Data analysis	Statistical analyses were performed in Graphpad Prism 9.<br>IF microscopy images were processed in Zen 3.1 (blue edition).<br>Cryo-EM data processing was performed in cryoSPARC v3.2.0 and the model was refined in Phenix v1.15.2-3472.<br>Bioinformatics analysis was performed using Plascad v1.17, BLAST v2.7.1, Python Toolkit "ETE" v3.0, RAxML v8.2.8 and Microreact v157 (www.microreact.org).<br>Molecular graphics and analyses were performed in ChimeraX-1.2.5<br>Multiple sequence alignments were performed in Clustal Omega. Alignments and phylogenetic trees were generated in Jalview 2.11.2.2

For manuscripts utilizing custom algorithms or software that are central to the research but not yet described in published literature, software must be made available to editors and reviewers. We strongly encourage code deposition in a community repository (e.g. GitHub). See the Nature Portfolio guidelines for submitting code & software for further information.

Data

Policy information about availability of data

All manuscripts must include a data availability statement. This statement should provide the following information, where applicable:

- Accession codes, unique identifiers, or web links for publicly available datasets
- A description of any restrictions on data availability
- For clinical datasets or third party data, please ensure that the statement adheres to our policy

The data supporting the findings in this study are provided within the manuscript and Supporting Information. Accession IDs of published sequences for reference plasmids and genomes are listed in the Methods. The coordinates and structure factors of the TraN-OmpK36 complex have been deposited to the Protein Data Bank and Electron Microscopy Data Bank with ID codes 7SZI and 25677 respectively. Source data are provided with this paper.

Field-specific reporting

Please select the one below that is the best fit for your research. If you are not sure, read the appropriate sections before making your selection.

☒ Life sciences ☐ Behavioural & social sciences ☐ Ecological, evolutionary & environmental sciences

For a reference copy of the document with all sections, see nature.com/documents/nr-reporting-summary-flat.pdf

Life sciences study design

All studies must disclose on these points even when the disclosure is negative.

Sample size	No statistical methods were used to determine sample size. All assays were performed in biological triplicate as is standard in the field and the data obtained had passed normality tests prior to further statistical analyses.
Data exclusions	No data were excluded in the final analyses
Replication	All selection-based conjugation experiments were performed in biological triplicate. Real-time conjugation system (RTCS) assays were performed in technical and biological triplicate. All attempts at replication were successful.
Randomization	Randomization was not performed as it is not standard practice in the field.
Blinding	Blinding is not relevant to this study as all results (CFU counts and GFP emission values) could be obtained objectively. No subjective measurements were recorded in this study.

Reporting for specific materials, systems and methods

We require information from authors about some types of materials, experimental systems and methods used in many studies. Here, indicate whether each material, system or method listed is relevant to your study. If you are not sure if a list item applies to your research, read the appropriate section before selecting a response.

Materials & experimental systems

n/a	Involved in the study	
☐	☒	Antibodies
☒	☐	Eukaryotic cell lines
☒	☐	Palaeontology and archaeology
☒	☐	Animals and other organisms
☒	☐	Human research participants
☒	☐	Clinical data
☒	☐	Dual use research of concern

Methods

n/a	Involved in the study	
☒	☐	ChIP-seq
☒	☐	Flow cytometry
☒	☐	MRI-based neuroimaging

Antibodies

Antibodies used	Custom rat polyclonal anti-Klebsiella pneumoniae pKpQIL pilus: Thermofisher used at 1:100 for immunofluorescence microscopy. Donkey polyclonal anti-Rat IgG (H+L) conjugated to Alexa Fluor 488: Jackson Immunoresearch Cat# 712-546-150 used at 1:1000.
Validation	The custom rat polyclonal anti-KP pili antibodies were validated by immunofluorescence microscopy. It can be seen to bind specifically to the conjugative pilus expressed off a derepressed variant of pKpQIL (shown in Fig 1e).
