# Supplementary figures and images for: Mating pair stabilization mediates bacterial conjugation species specificity
Source: Nat Microbiol. 2022 Jun 13;7(7):1016–27. doi: 10.1038/s41564-022-01146-4 (PMC9246713; doi:10.1038/s41564-022-01146-4)

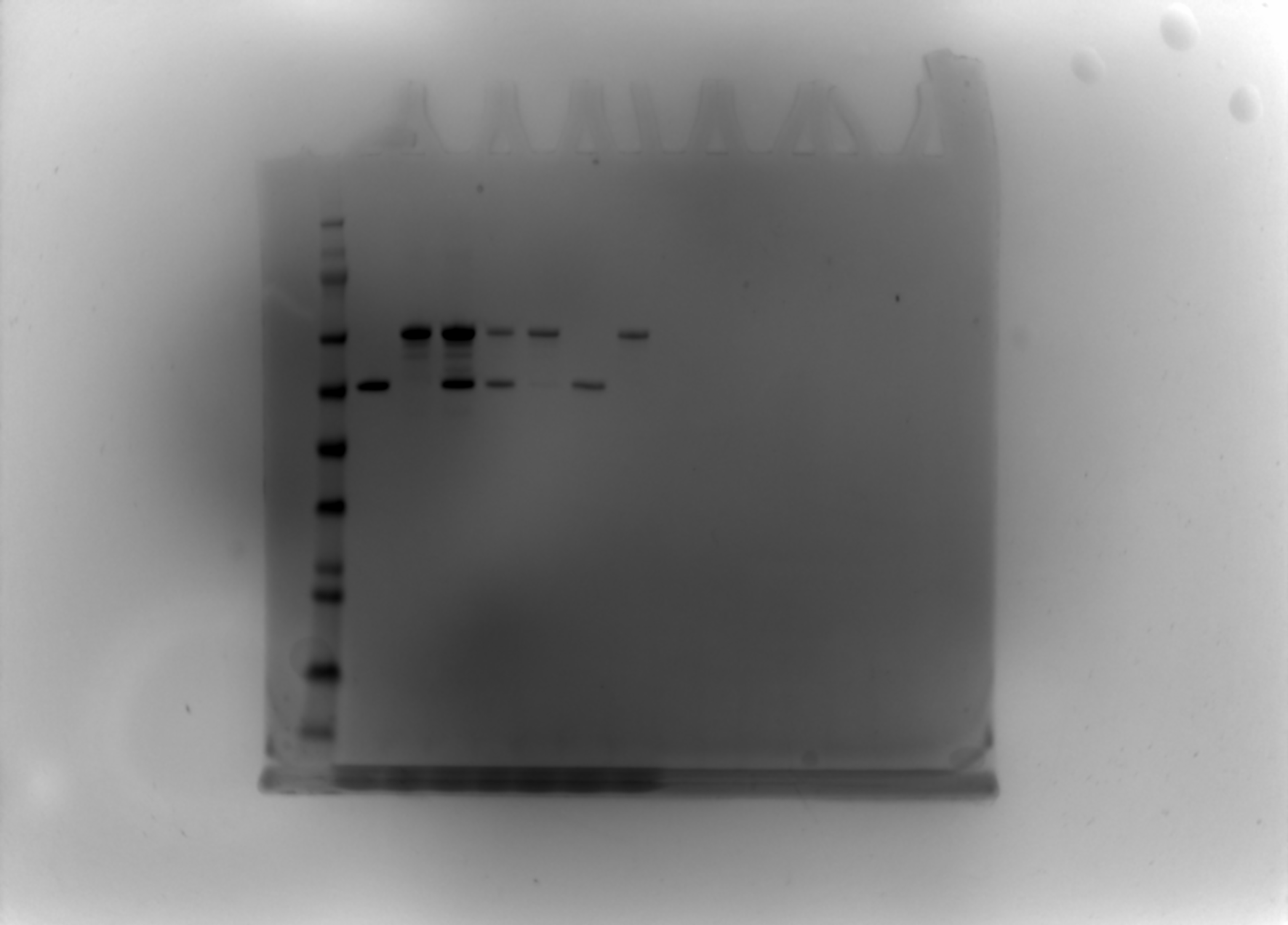

Supplement: Supplementary file 13 — Unprocessed Coomassie-stained SDS–PAGE gel. [file 41564_2022_1146_MOESM13_ESM.tif]
